# Stacking Order-Mediated Spin-State Modulation in Iron Phthalocyanine Covalent Organic Frameworks Enables Efficient Oxygen Reduction Reaction

**DOI:** 10.1021/acsami.5c19809

**Published:** 2026-01-23

**Authors:** Yun Li, Md. Samim Hassan, Desui Chen, Yuxuan Wu, Xin Zhao, Arsenii S. Portniagin, Haochen Liu, Shixun Wang, Penghui Ren, Ying Zhao, Andrey L. Rogach

**Affiliations:** † Department of Materials Science and Engineering, 53025 City University of Hong Kong, 83 Tat Chee Avenue, Kowloon, Hong Kong S.A.R. 999077, P. R. China; ‡ Department of Chemistry, 53025 City University of Hong Kong, 83 Tat Chee Avenue, Kowloon, Hong Kong S.A.R. 999077, P. R. China; § Department of Materials Science and Engineering, Hainan University, 58 Renmin Avenue, Haikou 570000, P. R. China; ∥ 639309 Shandong Laboratory of Advanced Materials and Green Manufacturing, Yantai 264000, P. R. China

**Keywords:** zinc-air battery, iron phthalocyanine, covalent organic framework, stacking order, spin-selected electron transport

## Abstract

Covalent organic frameworks (COFs) such as iron phthalocyanine (FePc) have been considered as potential electrocatalysts. Herein, we provide important insights into modulating the intrinsic activity of FePc COFs for the oxygen reduction reaction (ORR) by adjusting their stacking configuration. The eclipsed, AA-stacked and the staggered, AB-stacked FePc COF configurations were obtained via adjusting the interlayer interaction forces. Electrochemical studies reveal that the AA-stacked FePc COF exhibits a half-wave potential of 0.856 V vs RHE, which is 0.195 V higher than that of the AB-stacked FePc COF. The assembled zinc-air battery, using AA-stacked FePc COF as the cathode, demonstrates a high cell voltage of 1.64 V vs Zn^2+^/Zn alongside with a superior specific capacity of 935.79 mA h^–1^g_Zn_^–1^. The upshift in the valence band center and the high effective magnetic moment in the eclipsed, AA-stacked FePc COF suggest that the states are occupied at high energy levels, indicating a high-spin state of Fe. Density functional theory calculations suggest that the long-range spin channels aligned with iron columns in the eclipsed, AA-stacked FePc COF facilitate the spin-selective charge transport through interlayer band dispersion. The mechanism associated with the high-spin state of Fe promotes the cleavage of the *OO and *OOH intermediates, accelerating the ORR kinetics. Our study reveals that the stacking order of FePc COFs is important for modulation of the charge transfer and electron spin states, showing how to control the spin electronic characteristics of COFs through the stacking configuration-dependent interlayer interactions.

## Graphical abstract

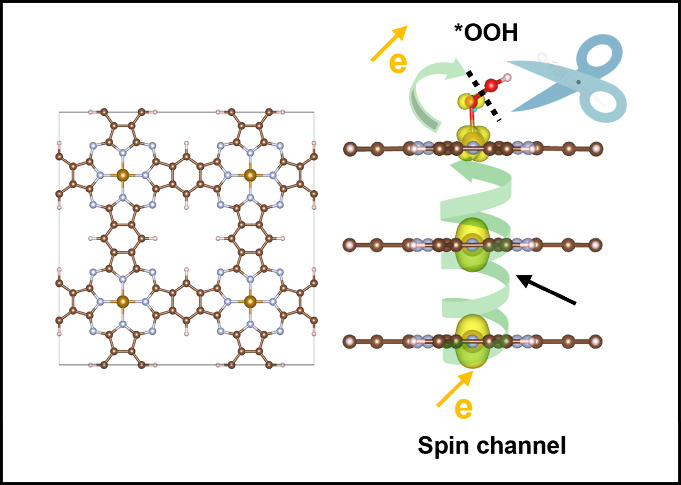

## Introduction

Covalent organic frameworks (COFs) are cross-linked polymers integrating various organic building units into ordered two-dimensional (2D) or three-dimensional (3D) networks, which have been considered for a variety of applications, particularly in heterogeneous catalysis.^1–3^ Oxygen reduction reaction (ORR) plays an important role in energy conversion processes, such as in fuel cells and zinc-air batteries. However, sluggish kinetics of this reaction significantly impedes its large-scale applications.^4^ Iron phthalocyanine (FePc) is considered as a promising alternative to the commercial electrocatalyst for ORR, i.e., platinum on carbon (Pt/C).^5–7^ Its catalytic activity arises from the square-planar Fe–N_4_ moiety featuring unsaturated low-coordination environment that provides adequate adsorption space in the axial direction, while the strong chelation between Fe and isoindole contributes to its high chemical and thermal stabilities.^8,9^

Oxygen reduction from triplet O_2_ to singlet H_2_O is a spin-forbidden process. It requires energy for spin orientation change during the four-electron transfer process, resulting in an overpotential of ∼ 0.4 V.^10^ In FePc, square-planar coordination causes the splitting of degenerate *d* orbitals of Fe (II) into *a*_1*u*_(*d*_*x* ^2^–*y* ^2^_), *e*_*g*_(*d*_*xz*_, *d*_*yz*_), *a*_1*g*_(*d*_*z* ^2^_), and *b*_2*g*_(*d*_*xy*_), leading to an intermediate spin for the *d* electrons (spin triplet, *S* = 1). This intermediate state facilitates the oxygen capture through orbital interactions and spin–orbital coupling.^11,12^ However, the difficulty in O=O dissociation on FePc limits its ORR activity as unpaired electrons in the *d* orbitals primarily aligned on the *z* axis often cause an end-side adsorption.^13^ Additionally, FePc undergoes a through-space pathway mechanism for charge transport, characterized by axial π-π stacking of its planar conjugated structure.^14^ Its in-plane charge transport is limited by the small range of a single π-conjugated phthalocyanine molecule.^15^

Constructing continuous conjugated π-bonds by incorporating FePc as blocks into a COF can enhance the in-plane delocalization and the interlayer interaction.^16^ It has been reported by Yu et. al^17^ that the stacking order can adjust the density of states (DOS) near the Fermi energy, which governs the heterogeneous charge-transfer rate between the electrocatalyst and the reactants. They found out that the kinetic rate of the redox couple (Ru­(NH_3_)_6_^3+/2+^ is accelerated by the interlayer moiré twist in bilayer graphene, relying on the increased DOS concentrated on the flat band formed by the hybridization between adjacent Dirac cones. An orderly spin–lattice is beneficial for optimal quantum spin exchange interactions, which not only acts as a spin filter for electron transfer but also helps to modulate the binding energies of oxygenated reactants.^18,19^ It was demonstrated that the magnetic structure of organic frameworks can be modulated through switching the stacking configuration.^20^ For example, Feng and co-workers^20^ observed a longer spin relaxation time in the staggered stacked Ni_3_(HATI)_2_ 2D frameworks (HATI = 2,3,7,8,12,13-hexaiminotriindole). Even though some studies demonstrated ways to adjust the stacking order of COFs, most of those utilized the steric hindrance effects by grafting a large-volume side group.^21^ This resulted in a great expansion of the interlayer spacing and thus influenced the interlayer electron transfer.

In this study, we demonstrate the precise switching of stacking behavior in FePc COFs by adjusting the interlayer interaction force and investigate the significant role in tuning both the valence state and the electron spin states of these materials. FePc COFs were synthesized through the quadruple intermolecular nucleophilic annulation of 1,2,4,5-tetracyanobenzene. The eclipsed, AA-stacked and the staggered, AB-stacked FePc COF configurations were obtained depending on the precursor amount. Electrochemical measurements indicate that the AA-stacked FePc COF achieves a high ORR activity with a half-wave potential of 0.865 V vs RHE. The fabricated zinc-air battery delivers an extremely high cell voltage of 1.64 V vs Zn^2+^/Zn and a high specific capacity of 935.79 mA h^–1^g_Zn_^–1^. Experimental and density functional theory (DFT) studies reveal that the stacking configuration-dependent modulation of the electron spin state of FePc COFs significantly influences their ORR performance. The upshift of the valence band center and the high effective magnetic moment indicate the state occupation at the high energy level and the high-spin state in the eclipsed AA-stacked FePc COF. Furthermore, the interlayer band dispersion reveals that a long-term spin channel exists along the iron column in the eclipsed, AA-stacked FePc COF. This promotes spin-selective transport of electrons and enhances the high-spin state at the Fe site, facilitating spin–orbital coupling between the active Fe site and the oxygenated intermediates. As a result, this interaction aids penetration of high-spin electrons into the antibonding π* orbital of the adsorbed oxygen molecule, promoting the cleavage of *O=O and *O–OH intermediates and thus facilitating ORR.

## Results and Discussion

FePc COFs were synthesized via the quadruple intermolecular nucleophilic annulation reaction of 1,2,4,5-tetracyanobenzene, where ferrous chloride (FeCl_2_) and 1,8-diazabicyclo­(5,4,0)­undec-7-ene (DBU) were introduced as precursors (Figure S1).^22^ We observed that varying the ratio of FeCl_2_ and DBU results in different types of stacking order and crystal structure of the FePc COFs, as discussed below. The FePc COFs synthesized with the same amount of DBU but different amounts of FeCl_2_ (35 and 150 mg) have been designated as COF-Fe-35 and COF-Fe-150. Meanwhile, the sample designated as COF-Fe-75 was synthesized using 75 mg of FeCl_2_ and 5 equivalents DBU. Powder X-ray diffraction (XRD) patterns indicate that COF-Fe-35 exhibits an amorphous structure (Figure S2), whereas COF-Fe-150 and COF-Fe-75 show well-established crystalline structures (Figure 1a). The observed XRD peaks of COF-Fe-75 at 2θ = 9.20, 18.60, 28.10, and 12.58° are attributed to the (100), (200), (300), and (110) planes, respectively, which is consistent with those of previously reported FePc COFs, indicating its eclipsed, AA-stacked structure.^23,24^ The intense XRD peak of COF-Fe-150 at 2θ = 10.95° is assigned to the (110) plane, suggesting that lattice expansion occurs.^25^ Pawley and Rietveld refinement of the observed XRD patterns further confirms the eclipsed stacking structure of COF-Fe-75 and the staggered stacking structure of COF-Fe-150, as indicated by small agreement factors in Table S1. Scanning electron microscopy (SEM) image demonstrates a flakelike morphology of COF-Fe-150 (Figure 1b). High-resolution transmission electron microscopy (HRTEM) image in Figure 1c indicates a lattice spacing of 7.30 Å in this case, corresponding to the (110) plane. As for COF-Fe-75, its SEM image shows a similar flakelike morphology (Figure 1d). Its HRTEM image (Figure 1e) indicates a lattice spacing of 7.01 Å corresponding to the (110) plane of the eclipsed-stacked FePc COF. Thus, HRTEM indicates that in the staggered configuration, the lattice along the (110) plane slightly expands from 7.01 to 7.30 Å, which is also corroborated by XRD data. This expansion is attributed to the interlayer steric effects of FePc COF due to its staggered, AB-stacked configuration.^21^ As shown in the atomic force microscopy (AFM) image, COF-Fe-75 has a platelike morphology with a thickness of ∼ 100 nm (Figure 1f), which should facilitate the out-of-plane charge transfer through interlayer dispersion.^26^ The elemental mapping indicates the uniform spatial distribution of C, N, and Fe elements in COF-Fe-75 (Figure 1g). The characteristic Raman shifts in Figure 1h for the FePc-subunit within the wavenumber range of 1000 ∼ 1550 cm^–1^ demonstrates the in-plane macrocycle vibration and in-plane C–H bending.^27–29^ Fourier transform infrared (FTIR) spectroscopy further confirms the phthalocyanine structure of the synthesized FePc COFs (Figure S4).^30^

**Figure 1:**
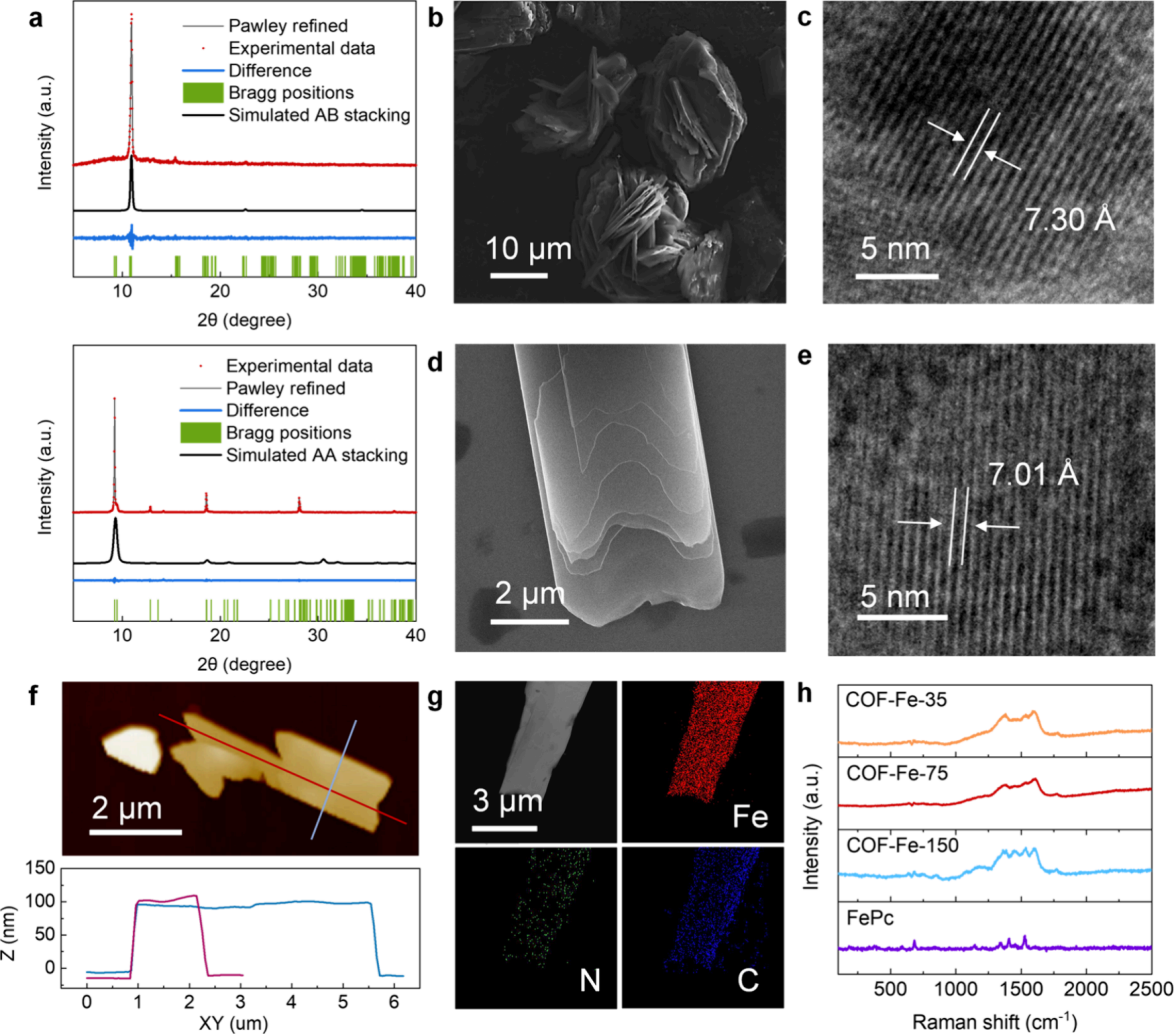
Characterization of the FePc-based COFs synthesized in this work. (a) Powder XRD patterns of AB-stacked COF-Fe-150 (top) and AA-stacked COF-Fe-75 (down). (b) SEM image and (c) HRTEM image of COF-Fe-150. (d) SEM image and (e) HRTEM image of COF-Fe-75. (f) AFM image with the height profiles of COF-Fe-75. (g) Elemental mappings for C, N, and Fe in COF-Fe-75. (h) Raman spectra of COF-Fe-35, COF-Fe-75, COF-Fe-150 and FePc.

To determine how the precursors (FeCl_2_ and DBU) influence the stacking behavior during the formation of FePc COFs, we varied their amount and analyzed the relationship between the ratio of precursors and the resulting stacking mode in FePc COFs. When the amount of FeCl_2_ is kept constant and the amount of DBU increases, we observe a transition from a staggered, AB-stacking mode to an eclipsed, AA-stacking mode (Figure S5). Conversely, increasing the amount of FeCl_2_ while keeping the amount of DBU constant results in an inverse transition from AA-stacking to AB-stacking (Figure S6). We also observe that the stacking mode of FePc COFs is closely related to the ratio (mg: μL) of FeCl_2_ and DBU. When this ratio is less than 0.15, an AA-stacking mode is predominant in the formation of FePc COF. In contrast, when the ratio exceeds 1.5, the AB-stacking mode becomes dominant. We infer that during the assembly process of FePc COFs, in addition to Fe atoms centrally located within 4 isoindole subunits, the remaining Fe­(II) cations coordinate with two unsaturated N atoms linking the isoindole units, which are separately located at the adjacent FePc COF monolayer. This coordination has a significant influence on the assembly of the FePc COF, leading to a staggered AB-stacking configuration. On the other hand, inadequate Fe cations prevent proper interlayer coordination owing to its priority to chelate with the formed phthalocyanine unit within the monolayer, resulting in the strong interlayer π-π interaction-driven eclipsed AA-stacking configuration in the formed FePc COF.^31,32^ This is also evident from the higher Fe content of 16.2 wt % in COF-Fe-150 as compared to 11.2 wt % in COF-Fe-75 and 9.2 wt % in FePc molecules, as determined by inductively coupled plasma spectrometry. Also, the effects of temperature and reaction time have been studied, as demonstrated in Figures S7 and S8, respectively. Generally, a higher reaction temperature and an extended synthesis time ensure the nucleation and growth of FePc COF, leading to the well-defined crystalline structure.

Electrocatalytic performance of the three synthesized FePc COFs was evaluated using a three-electrode system with a rotating disk electrode in an O_2_-saturated 0.1 M KOH. Here, we also introduce the FePc powder as an additional reference sample to distinguish whether the extended conjugation in FePc COFs influences the ORR performance. Linear sweep voltammetry (LSV) measurements (Figure 2a) reveal that COF-Fe-75 exhibits a superior ORR activity with a half-wave potential of 0.865 V vs RHE compared to FePc (0.738 V), COF-Fe-35 (0.713 V), and COF-Fe-150 (0.661 V). Its ORR performance also favorably compares with various other metal-coordinated COF-based electrocatalysts reported in the literature (Figure S10). Lower Tafel slope of 44.3 mV dec^–1^ observed for COF-Fe-75 (Figure S11) indicates a faster ORR kinetic process. We also studied the ORR performance for various loadings of the COF-Fe-75 catalyst. As illustrated in Figure S12, increasing limited current densities are observed with the elevated loading of COF-Fe-75. Meanwhile, the half-wave potential demonstrates a positive relation with the loading, which increases from 0.857 to 0.892 V vs RHE when the loading changes from 0.26 to 0.72 mg cm^–2^. However, further increase of loading has less influence on the half-wave potential. This means that the ORR performance of COF-Fe-75 is comparable to the commercial Pt/C catalyst (0.894 V vs RHE, Figure S14). To investigate the mechanism by which FePc COFs catalyze the ORR, a rotating ring-disk electrode was employed to analyze the yield ratio of evolving H_2_O_2_. As shown in Figure 2b, there is almost no H_2_O_2_ produced by COF-Fe-75, which suggests that the ORR proceeds through a four-electron reduction mechanism.^33^ This is further verified by the electron-transfer number (*n* = 4.005) in this case, derived from the Koutecky–Levich plot (as shown in Figures S15 and S16). COF-Fe-150 and COF-Fe-35 exhibit H_2_O_2_ yield ratios of 7.6 and 3.7%, respectively, which indicates that these two electrocatalysts follow a partial two-electron transfer pathway; their electron-transfer numbers are indeed less than 4 (see Figures S17–S20).^34,35^ KSCN poisoning experiment determines the Fe–N_4_ moiety as the primary active site in COF-Fe-75 (Figure S21). A significant decrease of 58 mV in the half-wave potential is observed during continuous LSV scans until the eighth scan, and notably, addition of KSCN has a limited effect on the onset potential, which differs from the previous reported Fe–N–C electrocatalysts.^36^ This suggests the presence of active nitrogen sites that contribute to the ORR activity of the COF-Fe-75 catalyst.^37^ Accelerated degradation test was conducted to evaluate the stability of COF-Fe-75 under transient varying reduction potentials, which demonstrates a negligible activity decay with only a 10 mV increase in overpotential after 5000 cycles (Figure 2c). Chrono-potentiometric analysis shown in Figure S22 indicates that COF-Fe-75 retains 83.5% of its current after operating for 10 h at 0.6 V vs RHE. XPS (Figure S23) and XRD (Figure S24) analyses both verify the stability of COF-Fe-75 in catalyzing the ORR process. Additionally, the electrochemical performance of the oxygen evolution reaction (OER) was also measured in 1 M KOH for the four samples studied. Polarization curves in Figure 2d show that COF-Fe-75 has the lowest overpotential of 281 mV at the current density of 10 mA cm^–2^, which is lower than that of commercial RuO_2_ (296 mV, Figure S25). Chrono-potentiometric analysis further shows that COF-Fe-75 demonstrates a remarkable stability under oxidation potential as only a negligible increase of overpotential is observed after a 100 h stability test at 10 mA cm^–2^ (Figure 2e).

**Figure 2:**
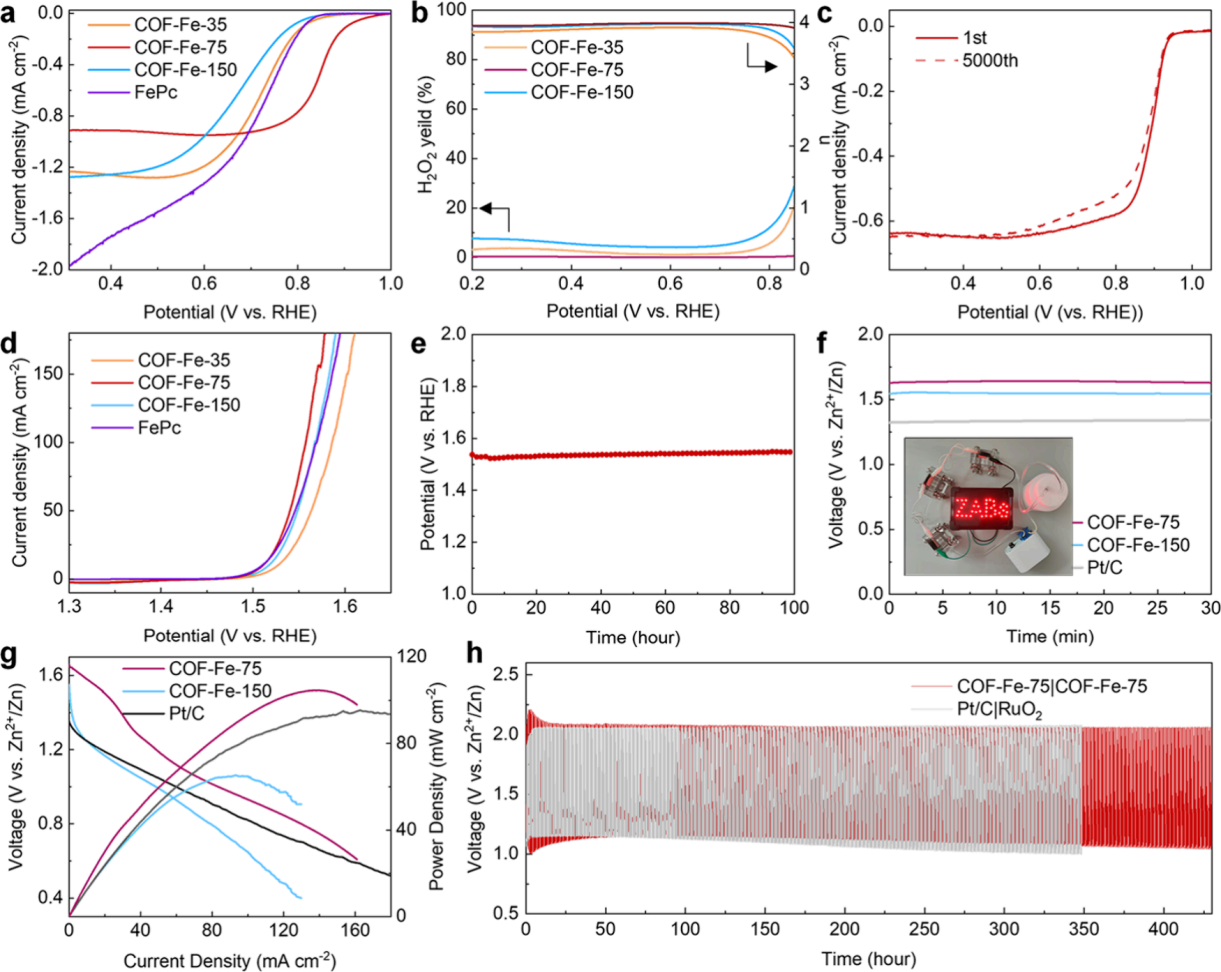
Electrochemical characterization of the synthesized FePc-based COFs. (a) Polarization curves of COF-Fe-35, COF-Fe-75, COF-Fe-150, and FePc in the ORR process. (b) Yield ratio of H_2_O_2_ and the calculated electron transfer number (*n*) for COF-Fe-35, COF-Fe-75, and COF-Fe-150 in the ORR process. (c) Polarization curves of COF-Fe-75 before and after 5000 cycles’ accelerated degradation test. (d) Polarization curves of COF-Fe-35, COF-Fe-75, COF-Fe-150, and FePc in the OER process. (e) Chrono-potentiometric curve of COF-Fe-75 at 10 mA cm^–2^. (f) Static open-circuit potential curves of COF-Fe-75, COF-Fe-150, and 20% Pt/C assembled zinc-air batteries; the inset is the photograph demonstrating an operational light-emitting diode board powered by the zinc-air battery with COF-Fe-75 as the cathode. (g) Polarization and power density curves of zinc-air batteries using COF-Fe-75, COF-Fe-150, and 20% Pt/C as cathodes. (h) Charge and discharge curves of COF-Fe-75|COF-Fe-75 (red) and Pt/C|RuO_2_ (gray) zinc-air batteries.

Encouraged by the results from the electrochemical tests, we assembled aqueous zinc-air batteries using synthesized FePc COFs as cathodes. Open-circuit potential was first tested with varying times, as illustrated in Figure 2f. Two batteries featuring COF-Fe-75 and COF-Fe-150 as the active phases exhibit a relatively high cell voltage of 1.64 V vs Zn^2+^/Zn and 1.55 V vs Zn^2+^/Zn, respectively. These values are close to the cell voltage under thermodynamic equilibrium and significantly higher than for the zinc-air battery using commercial 20% Pt/C electrode (1.33 V vs Zn^2+^/Zn).^38^ Polarization curves indicate that COF-Fe-75 with two discharge platforms has a higher power density of 105 mW cm^–2^, while 20% Pt/C reaches a power density of only 95 mW cm^–2^ (Figure 2g). The effect of COF-Fe-75 loading and addition of Nafion 117 on the power density of an assembled zinc-air battery has been investigated. Figure S26 reveals a positive correlation between the loading of the catalyst and the power density. Lower amount of Nafion 117 contributes to increased power density. A high power density of 251.2 mW cm^–2^ is achieved when the volume ratio of methanol and Nafion is 19:1, where methanol was used as the solvent to disperse the electrocatalyst. We also measured the discharge curves to calculate the specific capacity of FePc COFs, as illustrated in Figure S27. COF-Fe-75 demonstrates a specific capacity of 935.79 mA h^–1^g_Zn_^–1^, which is higher than those of COF-Fe-150 (847 mA h^–1^g_Zn_^–1^) and commercial 20% Pt/C (731.30 mA h^–1^g_Zn_^–1^). Based on the redox activity of FePc COFs, we then assessed the charge–discharge cyclic performance of the respective zinc-air batteries. There are several cycles with a decline in activity at the beginning; however, the performance stabilizes shortly thereafter, remaining consistent without deterioration for over 400 h (Figure 2h). At the same time, a significant deterioration in activity is observed for the noble metal-assembled cells (Pt/C and RuO_2_), highlighting the superior stability of FePc COFs used as a cathode in the zinc-air battery (Figure 2h). As shown in the inset of Figure 2f, the assembled zinc-air battery based on COF-Fe-75 can successfully power a light-emitting diode board.

To understand the reasons behind the variation in the ORR activity among the synthesized FePc COFs with different stacking orders (AA vs AB), we first employed electrochemical double-layer capacitance and electrochemical impedance spectroscopy, allowing us to analyze how the electrochemical active area and charge-transfer resistance influence the performance of FePc-based COFs. As shown in Figure S32, COF-Fe-75 with AA-stacking order has a relatively low active area among the FePc COFs, which aligns with the limited current observed from the LSV curves. This can be attributed to the quasi-2D structure of FePc COFs compared with the other electrocatalysts based on 3D porous carbon materials.^39^ At the same time, it exhibits the lowest charge-transfer resistance, indicating a high intrinsic activity of the AA-stacked FePc COF (Figure S33).^40^

Electronic band structures of FePc COFs were investigated to better understand their enhanced reducibility to oxygen. The band gaps of the synthesized FePc COFs with different stacking orders were derived from the ultraviolet–visible adsorption spectra via the Kubelka–Munk equation (Figure S34).^41^ Both COF-Fe-150 and COF-Fe-75 demonstrate narrower band gaps of 1.50 and 1.53 eV, respectively, as compared to FePc which has a band gap of 1.81 eV (Figure 3a). This narrowing is attributed to the continuous in-plane and out-of-plane delocalizations within the ordered framework.^42^ The valence band spectra of FePc COFs, measured by ultraviolet photoelectron spectroscopy (UPS), are depicted in Figure 3b, revealing an increased density of occupied states near the Fermi energy for both COF-Fe-75 and COF-Fe-150 as compared to FePc. Notably, there is a 0.30 eV upshift of the valence band center in COF-Fe-75, indicating an increase in occupancy at high energy levels. Additionally, as illustrated in Figure 3c, the extension of the conjugation in FePc COFs contributes to the upward shift of the Fermi level for COF-Fe-75 (−4.44 eV) and COF-Fe-150 (−4.55 eV) compared to FePc (−5.27 eV). This shift enhances their ability to donate electrons and improve their ORR activity.^43,44^

**Figure 3:**
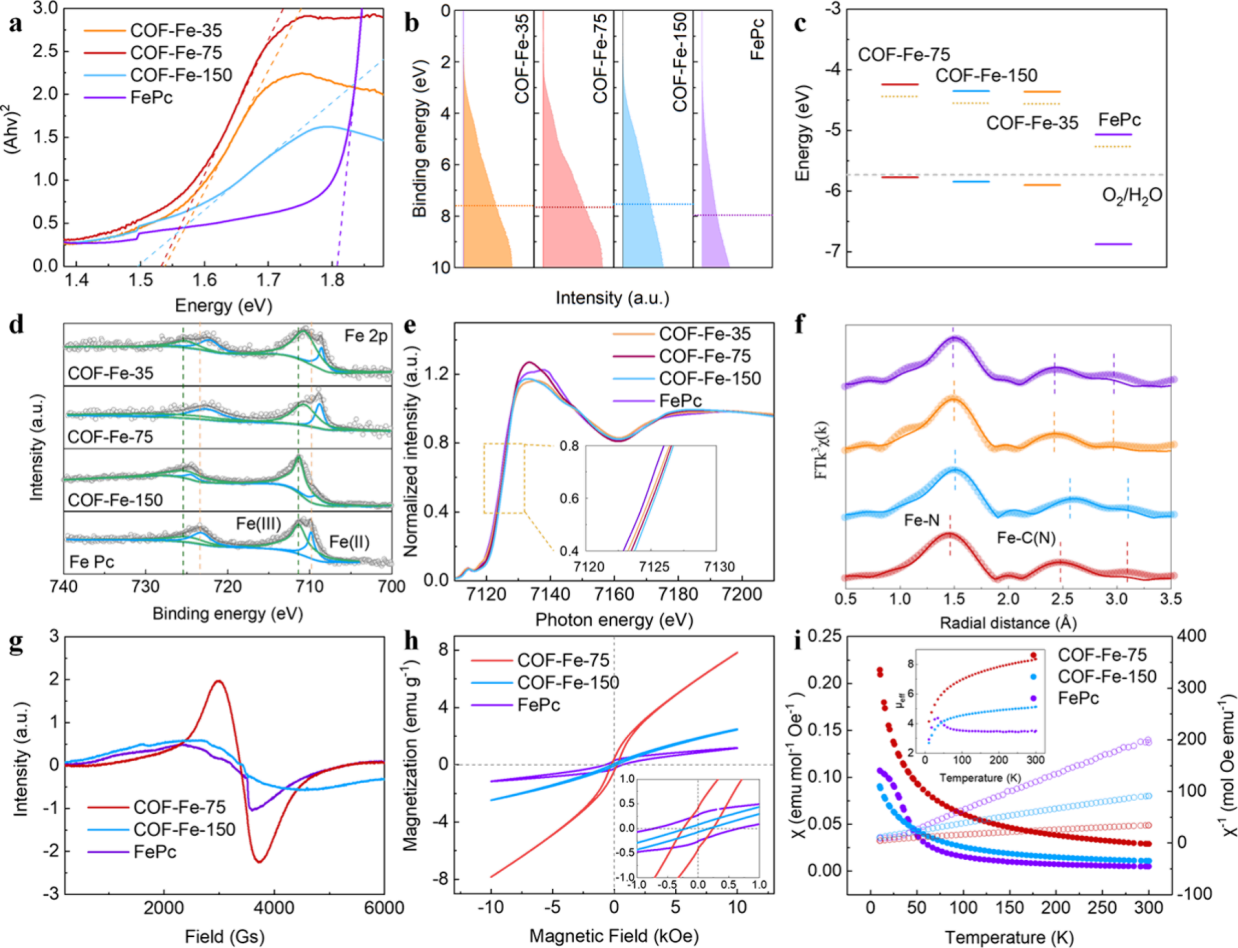
Electronic characteristics of the synthesized FePc-based COFs. (a) Tauc plots derived from UPS, (b) valence band spectra, (c) energy band structure, (d) high-resolution XPS spectra of Fe 2p orbitals, (e) XANES spectra, (f) Fourier transform fitting curves of the k^3^-weighted Fe K-edge EXAFS spectra, and (g) EPR spectra of COF-Fe-75, COF-Fe-150, and FePc. The enlarged selected region in Figure 3e corresponding to the yellow square area is shown in the inset. (h) Magnetic hysteresis loops measured at 2 K as well as (i) temperature-dependent magnetic susceptibility plots under a magnetic field (1 T) of COF-Fe-75 and COF-Fe-150, where the inset shows the temperature-dependent effective magnetic moment.

We then investigated the local electronic characteristics of the synthesized FePc COFs, focusing on how they depend on the stacking orders, using X-ray photoelectron spectroscopy (XPS) and fine structure measurements. As shown in Figure 3d, characteristic Fe­(II) and Fe­(III) 2p peaks are present in FePc, COF-Fe-75, and COF-Fe-150. Compared to FePc, the deconvoluted Fe­(II) 2p peak in COF-Fe-75 exhibits a negative shift from 709.9 to 708.8 eV. Similarly, the peak in COF-Fe-150 also shows a slight negative shift of 0.5 eV. This indicates that the extension of the conjugated region in COF-Fe-75 and COF-Fe-150 contributes to the negative shift of Fe (II) 2p peak, while the strong interlayer dispersion in the eclipsed Fe–N_4_ moieties in COF-Fe-75 leads to more electron accumulation on the Fe site compared with COF-Fe-150. The deconvoluted C 1s XPS spectra of COF-Fe-75 reveal the characteristic peaks for C=C, C–C, and C=N at 284.6, 285.2, and 286.2 eV, respectively (Figure S35).^45^ Deconvolution of the N 1s XPS spectra shows the coexistence of C=N–C and C=N–Fe in COF-Fe-75 (Figure S36), which also experience a negative shift compared with that of FePc.^46^ This suggests that the electronic state changes through the extension of the conjugation region and the variation of the stacking order of FePc COF sheets. X-ray absorption near-edge structure (XANES) spectra (Figure 3e) provide further insights into the coordination environment of the Fe site in eclipsed, AA-stacked COF-Fe-75 and staggered, AB-stacked COF-Fe-150. Similar pre-edge absorption indicates that Fe in FePc COFs exhibits an analogous coordination environment as in the case of the FePc reference sample. This is characterized by a square-planar and centrosymmetric Fe–N_4_ configuration featuring a fingerprint shoulder at 7114.14 eV, which is attributed to the 1s → 4p_*z*_ transition accompanied by simultaneous ligand-to-metal charge transfer.^47,48^ In comparison with the AB-stacked COF-Fe-150, Fe in the AA-stacked COF-Fe-75 is in a lower oxidation state, indicative of a larger accumulation of electrons at the Fe site. The least-squares extend X-ray absorption fine structure (EXAFS) fitting (Figure 3f) was performed with related parameters shown in Figure S37 and Table S3. It shows a shorter Fe–N bond length of 1.95 Å in the AA-stacked COF-Fe-75 compared to 2.01 Å in the AB-stacked COF-Fe-150 and 1.97 Å in the FePc reference sample. The scattering from the second-shell pyrrolic C in FePc COFs shows an increased distance compared with the FePc reference sample, suggesting that the pyrrole units are elongated along the axis of the Fe–N bond.^49^This is further evidenced from the extended scattering distance in the third-shell N bonded with isoindole. The distortion of phthalocyanine motifs in FePc COFs may derive from the pedal motion around rigid C=N double bonds.^50^ A similar X-ray absorption fine structure of the amorphous COF-Fe-35 also points out toward the conjugation-induced distortion of isoindole subunits. The wavelet transforms contour plots (Figure S38) show a maximum intensity at around 5 Å^–1^, indicating the similar Fe–N_4_ structure in the four samples studied.

To further investigate the electron spin configuration of FePc COFs with varying stacking orders, electron paramagnetic resonance (EPR) spectra, magnetic hysteresis loops (M-H), and temperature-dependent magnetic susceptibility (χ-T) were measured. As illustrated in Figure 3g, EPR spectra reveal a strong paramagnetic behavior in the eclipsed, AA-stacked COF-Fe-75, while the staggered, AB-stacked COF-Fe-150 exhibits weak para-magnetism. This indicates that COF-Fe-75 contains more unpaired electrons. The magnetic hysteresis phenomenon (Figure 3h) indicates the ferromagnetic properties of FePc, COF-Fe-75, and COF-Fe-150 at 2 K, where the saturation magnetization (3.324 emu g^–1^) and remanence (0.402 emu g^–1^) of COF-Fe-75 are higher than those of the FePc reference sample (1.186 and 0.271 emu g^–1^) and COF-Fe-150 (2.494 and 0.074 emu g^–1^). As shown in Figure 3i, χ-T plots show a nearly linear temperature-independent para-magnetism for FePc, COF-Fe-75, and COF-Fe-150. The derived χ^–1^ versus T plots could be well fitted by the Curie–Weiss law, indicating an increase of the Curie temperature for the FePc COFs.^51^ The calculated effective magnetic moments (μ_eff_) for FePc, COF-Fe-150, and COF-Fe-75 at room temperature (inset of Figure 3i) are 3.47, 5.12, and 8.33 μ_B_, respectively. This suggests that COF-Fe-75 exhibits a high-spin state of electrons in its *d* orbitals, which is attributed to strong interlayer interactions. These interactions may facilitate the ability of these *d* electrons to penetrate the antibonding π-orbital of oxygen, thereby enhancing the ORR process.^52^ In contrast, the lower-spin state observed in COF-Fe-150 makes it difficult for electron transfer to occur between the Fe sites and the oxygenated intermediates. As a result, COF-Fe-150 exhibits poor ORR activity despite having a higher Fe content than COF-Fe-75. Though the observed upshift of the valence band center in COF-Fe-75 may hinder the electron transfer during the OER process, the high-spin state enhances the spin–orbital coupling with oxygenated intermediates. As a result, COF-Fe-75 demonstrates comparable OER activity with FePc, while the upshift of valence band center and the low-spin state in COF-Fe-150 leads to a relatively poor OER performance. Additionally, due to the extended conjugated region, efficient charge carrier transfer occurs in COF-Fe-75, leading to its improved OER performance at high current density.

Interlayer interactions of FePc COFs were further investigated through DFT calculations to figure out how different stacking configurations influence the electron spin state of the Fe–N_4_ matrix and eventually lead to different adsorption behaviors of oxygenated intermediates in the ORR process. The DFT calculations were conducted using Vienna ab initio Simulation Package based on the Perdew–Burke–Ernzerhof method; computational details are provided in the Supporting Information. The simulated cell structures for the AA-stacked and AB-stacked FePc COFs are shown in Figure S39 and Table S4. The reaction pathways of ORR, which include the reduction of O_2_ to*OO, *OOH, *O, *OH, and H_2_O, at the Fe–N_4_ site onto the (001) plane of both AA-stacked and AB-stacked FePc COFs are illustrated in Figure S40. To analyze the interlayer interaction within AA-stacked and AB-stacked FePc COFs, relative supercells were constructed with three single layers of FePc COF. The relative Gibbs free energy evolutions of the four-electron transfer mechanism are downhill at *U* = 0 V (Figure 4), implying that this process occurs spontaneously. Thermodynamic limiting potential, the highest potential at which the first two reaction steps (*OO + H_2_O + e → *OOH + OH^–^; *OOH + H_2_O + e → H_2_O_2_ + OH^–^) are downhill in free energy, was calculated to be 0.792 and 0.974 V for AB-stacked and AA-stacked FePc COF, respectively. This finding is consistent with the H_2_O_2_ measurements using a rotating ring-disk electrode system, which show that hydrogen peroxide is generated around the onset potential range. The first electron transfer step, *OO + H_2_O + e^–^ → *OOH + OH^–^, is considered as the sluggish step in the ORR process due to the high energy barrier associated with the cleavage of O=O bond.^53,54^ Notably, there is a clear drop of free energy in the AA-stacked FePc COF compared to AB-stacked FePc COF within this step (Figure 4a), contributing to their distinct ORR activity and selectivity of the four-electron transfer pathway.

**Figure 4:**
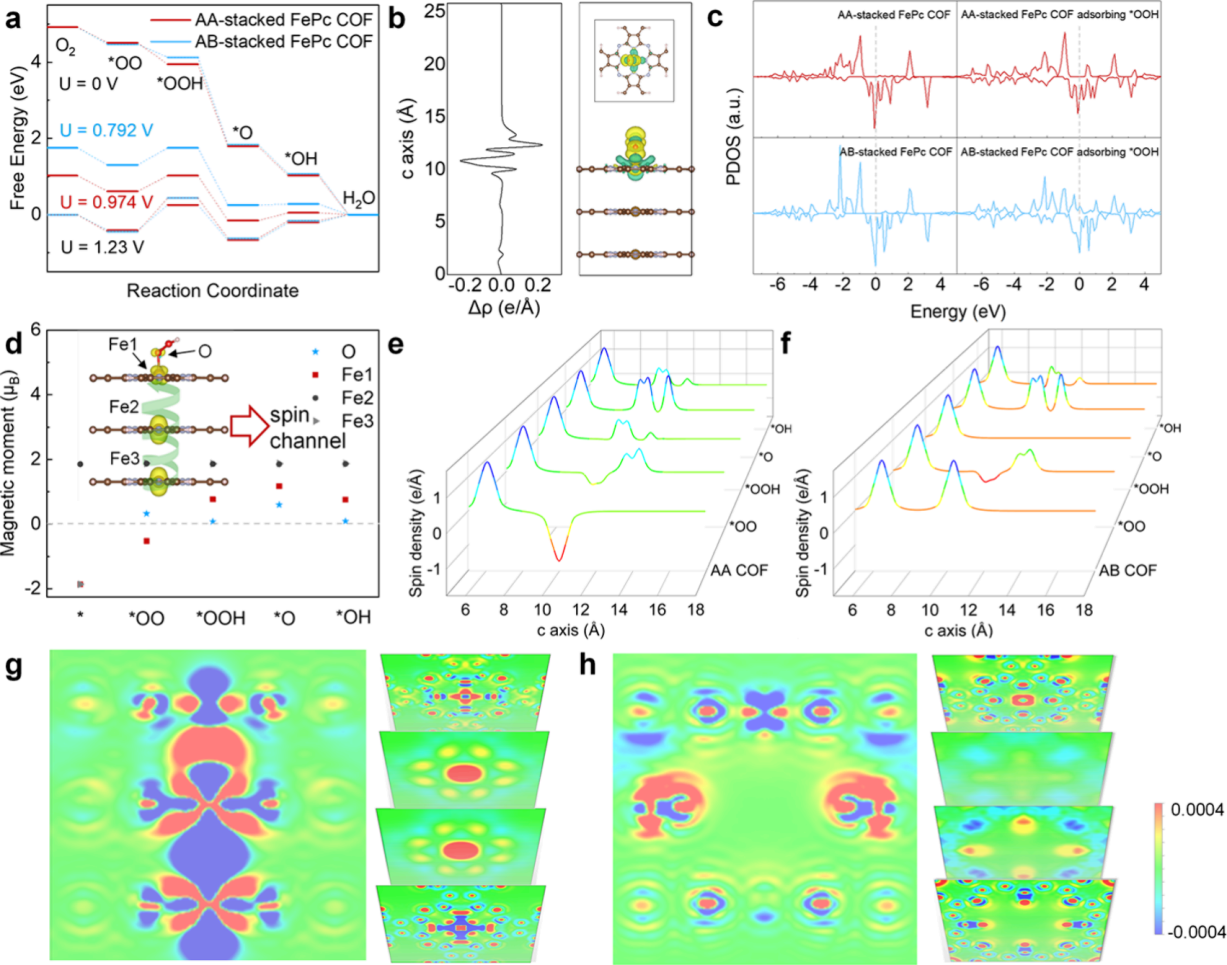
ORR mechanism analysis is based on DFT calculations. (a) Evolution of Gibbs free energies of ORR within AA-stacked and AB-stacked FePc COFs with and without (*U* = 0 V) an external potential. (b) Charge density difference in the AA-stacked FePc COF, where yellow and cyan regions denote the depletion and accumulation of electrons, respectively. (c) Projected DOS of the *d* orbitals of Fe within AA-stacked and AB-stacked FePc COFs with and without *OOH adsorbates. (d) Atomic magnetic moment of the AA-stacked FePc COF with different adsorbed oxygenated intermediates. The average spin density of states varied with the *c* axis for (e) AA-stacked and (f) AB-stacked FePc COFs when adsorbing oxygenated intermediates. The cross profiles of charge density difference parallel to the (010) plane (left) and separated cross figures parallel to the (001) plane (right) in (g) AA-stacked and (h) AB-stacked FePc COF, where the red and blue regions denote the depletion and accumulation of electrons, respectively.

To understand the factors leading to the observed drop in free energy based on the measured magnetic properties, we analyzed the spin–orbital interaction and electron transfer behavior between FePc COFs with AA- and AB-stacking arrangements and different adsorbed oxygenated intermediates. Both kinds of FePc COFs exhibit comparable ability to chemically adsorb oxygen molecules at the Fe–N_4_ moiety at the beginning of oxygen reduction (Figure 4a), which occurs spontaneously due to its exothermic nature. Our findings reveal electron transfer from the adsorbed oxygen to the Fe site in both kinds of FePc COFs (AA- and AB-stacked), as illustrated in the charge density difference profile (Figures 4b and S41). According to the ligand field effect, degenerated *d* orbitals of Fe chelated with four isoindole subunits are split into *a*_1*u*_(*d*_*x* ^2^–*y* ^2^_), *e*_*g*_(*d*_*xz*_, *d*_*yz*_), *a*_1*g*_(*d*_*z* ^2^_), and *b*_2*g*_(*d*_*xy*_), where four unpaired electrons are separately located at *a*_1*u*_, *a*_1*g*_, and *e*_*g*_ orbitals.^55,56^ Spin density, which is primarily distributed on the central Fe atom, as illustrated in Figure S42, facilitates hybridization between Fe­(*d*_*z* ^2^_ and *d*_*yz*_) and O_2_ (antibonding π*, mainly consisting of 2py and 2pz orbitals), favorably forming an end-side adsorption (Figure S43).^57^ The asymmetry of spin density (up and down) near the Fermi level indicates a high-spin state for Fe within the AA-stacked FePc COF, whose spin is stronger than that in the AB-stacking mode (Figure S44).^58^ As shown in Figure S44, the high-spin state remains in AA- and AB-stacked FePc COF when it absorbs the *OO intermediate, where the weak ligand field effect of *OO would lead to the distortion of the square-planar Fe–N_4_ configuration (Figure S45).^59^ In the case of AA-stacked FePc COF, the high-spin state is maintained when the adsorbed *OO is reduced to *OOH, resulting in a stronger binding strength of *OOH at the Fe site (Figure 4c).^19^ In this sense, a spin channel forms that connects the states below and across the Fermi level, propagating strictly in the spin-down domain. This spin channel facilitates the spin-allowed electronic coupling within the first two-electron transfer steps (*OO + H_2_O + e^–^ → *OOH + OH^–^; *OOH + e → *O + OH^–^). In this way, the cleavage of the *O=O and *O–OH bonds is stimulated, and the energy required for changing the spin orientation during electron transfer is almost exempted. In contrast, the high symmetry of DOS below the Fermi level indicates the low-spin state for Fe in the AB-stacked FePc COF when it adsorbs the *OOH intermediate, resulting in difficulty in this cleavage. Notably, no such spin channel forms in the AB-stacked FePc COF, leading to weaker binding of the *OOH intermediate and the easier generation of H_2_O_2_.^60^ Additionally, a higher *d*-band center of the Fe site in the AA-stacked FePc COF compared to the AB-stacked case indicates an enhanced electron donation ability, implying an improved capacity for the electron transfer to oxygenated intermediates (Figure S46).

We also calculated the atomic magnetic moments of Fe atoms in the AA-stacked FePc COF both with and without adsorbing oxygenated intermediates, denoted as Fe1, Fe2, and Fe3 in Figure 4d. Besides the slab model, we calculated the atomic magnetic moments of Fe atoms within a periodic supercell (Figure S47). Atomic magnetic field exists along the Fe column and acts as a long-term spin channel to facilitate spin-selected electron transport. Simultaneously, van der Waal interactions, especially interlayer out-of-plane dispersion, were calculated on the basis of Grimme DFT-D3 method.^61–63^ This interlayer dispersion is tightly associated with the charge transfer along the *z* axis of FePc COFs following a through-space mechanism, which can be visualized through the cross-section figure of charge density difference parallel to the (010) plane and separated cross-section figures parallel to the (001) plane by a certain distance between the two adjacent single FePc COF layers, as illustrated in Figure 4g,h. Here, the out-of-plane dispersion in the AA-stacked FePc COF mainly depends on the alignment of linear channels within the iron columns, while in the AB-stacked FePc COF, multiple zigzag channels for dispersion are formed. This configuration allows the AA-stacking arrangement to provide a long-term spin charge transfer channel along with the iron column, which facilitates both charge transfer and spin filter. Consequently, the penetration of unpaired electrons into the antibonding π orbital in oxygenate intermediates becomes strengthened. That is why an obvious decrease of the magnetic moment, from 1.86 to 0.77 μ_B_, is observed at the Fe1 site when adsorbing *OOH, reflecting the coupling of unpaired electrons between the Fe1 site in the AA-stacked FePc COF and the O site in the *OOH intermediate (Figure 4d). The average spin density of states as a function of position along the *c* axis for both AA-stacked and AB-stacked FePc COFs, as shown in Figure 4e,f, respectively, specifically illustrates the evolution of spin state change during the four-electron transfer pathway. These profiles indicate that there are nearly no unpaired electrons localized around Fe and O atoms in the AB-stacked FePc COF with the adsorbed *OOH intermediate as no such spin channel is formed in this case. It requires additional energy for the subsequent spin-forbidden reduction step (*OOH + e → *O + OH^–^). Thus, in comparison to the AB-stacked COF, the AA-stacked FePc COF exhibits a more thermodynamically favorable ORR process and a higher ORR activity, which is attributed to the spin-selected transport of electrons within the iron column.

## Conclusions

FePc-based COFs with eclipsed (AA) and staggered (AB) stacking order were synthesized through adjusting the interlayer interaction forces; they exhibited distinct electron spin characteristics. The spin channel formed along the iron column in the eclipsed (AA-stacked) FePc COF enhances its ORR activity compared to the staggered (AB-stacked) FePc COF. The eclipsed configuration achieves a high half-wave potential of 0.865 V vs RHE and a specific capacity of 935.79 mA h^–1^g_Zn_^–1^ in the assembled zinc-air battery. The high magnetic moment of the eclipsed FePc COF indicates the state occupation at high energy levels and the resulting high-spin state. DFT calculations reveal that a long-term spin channel exists in the iron column of the eclipsed AA-stacked FePc COF, which supports the spin-selective transport of electrons and facilitates the cleavage of *O=O and *O–OH bonds. However, additional energy for spin reorientation is required for the staggered, AB-stacked FePc COF due to the weakened spin state of the Fe site when oxygenated intermediates, particularly *OOH, are absorbed. This leads to a significant generation of hydrogen peroxide and a lower discharge voltage during the oxygen reduction process. Our study elucidates the feasibility of adjusting the stacking configuration of FePc COF to manipulate the spin state and charge transport behavior, offering insights into the relationship between spin characteristics and electrocatalytic activity.

## Experimental Section

### Chemicals

All chemicals were in analytical grade and were used without further purification. 1,8-diazabicyclo[5.4.0]­undec-7-ene, iron­(II) chloride, potassium hydroxide solution, zinc acetate, and Nafion 117 solution were purchased from Aladdin Scientific Corp. 1,2,4,5-Tetracyanobenzene was purchased from Macklin Inc. Ethylene glycol and dimethylformamide were purchased from the Sigma-Aldrich. Commercial 20% Pt/C and RuO_2_ electrocatalysts were purchased from Sinero Tech. Corp.

### Synthesis of FePc COFs

FePc COFs were synthesized according to the previous reported method with some modifications.^22^ To synthesize COF-Fe-75, 100 mg of 1,2,4,5-tetracyanobenzene and 75 mg of FeCl_2_ were added into a three-necked flask filled with the mixture of ethylene glycol and dimethylformamide (volume ratio = 9:1) under continuous stirring. After they were completely dissolved, 500 μL of 1,8-diazabicyclo[5.4.0]­undec-7-ene was slowly dropped in, and the solution immediately became dark green. The flask underwent degas–gas process three times on a Schlenk-line and then was gradually heated and kept at 180 °C for 24 h. When cooled down to the room temperature, the dark green products were collected by centrifugation. After washing with dimethylformamide, deionized water, and methanol, the final product COF-Fe-75 was dried in a vacuum oven at 40 °C for 24 h. COF-Fe-150 was synthesized in a similar manner, except using 150 mg of FeCl_2_ and 100 μL of 1,8-diazabicyclo[5.4.0]­undec-7-ene. COF-Fe-35 was synthesized by using 35 mg of FeCl_2_ and 100 μL of 1,8-diazabicyclo[5.4.0]­undec-7-ene.

### Characterization

Powder XRD patterns were recorded on a Bruker D2 Phaser XE-T X-ray diffractometer with Cu Kα radiation (λ = 0.154 nm) at a voltage of 40 kV and a current of 40 mA. Transmission electron microscopy (TEM) images were obtained on a JEM-2100F field emission electron microscope operated at 200 kV. SEM images were collected on a Thermo Scientific scanning electron microscope (Quattro S) operated at 20 kV. AFM measurements were performed on a Bruker Multimode 8 atomic force microscope. FTIR spectra were recorded on a PerkinElmer Spectrum Two FTIR spectrometer. Raman spectra were measured on a WITec Alpha300 Raman imaging microscope. XPS measurements were conducted on a Thermo Fisher ESCALAB250 spectrometer in ultrahigh vacuum using an Al Kα X-ray source, which was calibrated by C 1s binding energy (284.8 eV). X-ray absorption fine structure spectra for the Fe K-edge were measured on the BL14W1 line station of Shanghai Synchrotron Radiation Facility in the transmission mode. EPR spectra were collected on a Bruker EMXplus-6/1 spectrometer (Germany), where each measurement used 5 mg of the sample. Magnetic properties were evaluated on a DynaCool-9T system, where the magnetic hysteresis loop was measured within the magnetic field range from −1 to 1 T at 2 K, and temperature-dependent magnetic susceptibility plots were measured in the temperature range 300 K down to 10 K under magnetic field of 1 T.

### Electrochemical Measurements

The electrochemical performance of the ORR was investigated in an O_2_-satuated 0.1 M KOH solution using a three-electrode system with a rotating disk electrode (glassy carbon, 5 mm diameter) as the working electrode. Graphite plate and mercuric oxide electrode were used as the counter electrode and the reference electrode, respectively. 2.5 mg of the prepared electrocatalyst was dispersed into the mixture of 50 μL of Nafion 117 solution and 450 μL of methanol. After ultrasonication, 10 μL of the prepared electrocatalyst ink was dropped onto the working electrode. LSV curves were measured at a rotating speed of 1600 rpm within the potential range of 0.3 ∼ 1.23 V vs RHE and a scanning rate of 10 mV s^–1^. Cyclic voltammetry (CV) curves were measured at a potential range of 1.05 ∼ 1.15 V vs RHE with varying scanning rates to calculate the electrochemical active area. Electrochemical impedance spectra were collected under 0.76 V vs RHE in the frequency range from 1 × 10^5^ to 0.01 Hz.

The yield ratio of hydrogen peroxide was measured using the rotating ring-disk electrode; the scanning potential range for the disk electrode was set the same as for the LSV test, while the potential at the ring electrode was set as 1.165 V vs RHE. The electron transfer number (*n*) and the H_2_O_2_ yield ratio (H_2_O_2_%) were calculated by the following formula:

$$n=\frac{4I_{D}}{I_{D}+I_{R}/N}$$

$$H_{2}O_{2}\%=\frac{2I_{R}/N}{I_{D}+I_{R}/N}$$

where *I*_D_ and *I*_R_ are the Faradaic current at the disk electrode and the ring electrode, respectively, and *N* is the collection coefficient of H_2_O_2_ at the ring electrode.

The electron transfer number *n* during the ORR process was determined from the Koutecky–Levich plot, which involved measuring polarization curves at different rotating speeds of the working electrode:

$$\frac{1}{j}=\frac{1}{j_{L}}+\frac{1}{j_{K}}=\frac{1}{B\omega^{1/2}}+\frac{1}{j_{K}}$$

$$B=0.62\times 0.19625\times {nFC}_{o}D_{o}^{2/3}v^{-1/6}$$

Here *j*, *j*_L_, and *j*_K_ represent the measured current density, diffusion current density, and kinetic current density, respectively; ω is the rotating speed of the working electrode; *F* is the Faraday constant, *C*_o_ is the volume concentration of oxygen (1.2 × 10^–6^ mol mL^–1^), *D*_o_ is the diffusion coefficient for oxygen in 0.1 mol L^–1^ KOH, and v is the kinematic viscosity of the electrolyte (0.01 cm^2^ s^–1^).

### Assembly of Zinc-Air Batteries

2.5 mg of the synthesized electrocatalyst and 2.5 mg of carbon black were added into the mixture of 900 μL of methanol and 100 μL of Nafion 117 solution, and the ink was ultrasonicated for 30 min. For comparison, 2.5 mg of the commercial 20% Pt/C, 2.5 mg of RuO_2_, and 2.5 mg of carbon black were used to prepare the reference electrodes in a similar manner. The cathode was composed of three layers, nickel foam, hydrophobic layer, and carbon paper, where 800 μL of the ink was dropped onto the carbon paper within 2 × 2 cm^2^ area. Before the assembly of the zinc-air battery, the zinc plate was polished with a sandpaper to remove any trace of ZnO from the surface. The assembled batteries were tested in an alkali environment (6 M KOH and 0.2 M zinc acetate) under room temperature.

## Supplementary Material



## Notes

Experimental section; schematic illustration of the synthesis of eclipsed (AA-stacked) and staggered (AB-stacked) FePc COFs; powder XRD pattern of COF-Fe-35; Pawley and Rietveld refined lattice parameters for COF-Fe-75 (AA-stacking) and COF-Fe-150 (AB-stacking); SEM image of COF-Fe-35; FTIR spectra of COF-Fe-35, COF-Fe-75, COF-Fe-150, and FePc; determination of the half-wave potential from the LSV curves for COF-Fe-35, COF-Fe-75, COF-Fe-150, and FePc; comparison of the ORR activity; Tafel plots derived from ORR polarization curves for COF-Fe-35, COFFe-75, COF-Fe-150, and FePc; LSV curves; Koutecky-Levich plots of COF-Fe-75 derived from the LSV curves; chrono-amperometric response of COF-Fe-75 for continuous ORR; high-resolution XPS spectra; comparison of bifunctional COF electrocatalysts with COF-Fe-75 reported in this work; polarization and power density curves of zinc-air batteries; specific capacities of the assembled zinc-air batteries; CV curves; Nyquist plots; absorption spectra; high-resolution XPS spectra; Fe K-edge EXAFS analysis for FePc, COF-Fe-150, and COF-Fe-75; wavelet transform contour plots; simulated lattice parameters of eclipsed, AA-stacked FePc COF and staggered, AB-stacked FePc COF; four-electron transfer mechanism of ORR; distribution of spin density; PDOS for the d orbitals; and spin distribution and atomic magnetic moment of Fe sites (PDF)

## References

[1] Fang Q., Gu S., Zheng J., Zhuang Z., Qiu S., Yan Y. (2014). 3D microporous
base-functionalized covalent organic frameworks for size-selective
catalysis. Angew. Chem., Int. Ed. Engl..

[2] Lin S., Diercks C. S., Zhang Y. B., Kornienko N., Nichols E. M., Zhao Y., Paris A. R., Kim D., Yang P., Yaghi O. M., Chang C. J. (2015). Covalent organic
frameworks comprising cobalt porphyrins for catalytic CO(2) reduction
in water. Science.

[3] Wang J. C., Kan X., Shang J. Y., Qiao H., Dong Y. B. (2020). Catalytic Asymmetric
Synthesis of Chiral Covalent Organic Frameworks from Prochiral Monomers
for Heterogeneous Asymmetric Catalysis. J. Am.
Chem. Soc..

[4] Wang X., Zhong H., Xi S., Lee W. S. V., Xue J. (2022). Understanding
of Oxygen Redox in the Oxygen Evolution Reaction. Adv. Mater..

[5] Sorokin A. B. (2013). Phthalocyanine
metal complexes in catalysis. Chem. Rev..

[6] Chen Z., Jiang S., Kang G., Nguyen D., Schatz G. C., Van Duyne R. P. (2019). Operando
Characterization of Iron Phthalocyanine Deactivation
during Oxygen Reduction Reaction Using Electrochemical Tip-Enhanced
Raman Spectroscopy. J. Am. Chem. Soc..

[7] Snitkoff-Sol R. Z., Rimon O., Bond A. M., Elbaz L. (2024). Direct measurement
of the oxygen reduction reaction kinetics on iron phthalocyanine using
advanced transient voltammetry. Nature Catalysis.

[8] Yang S., Yu Y., Gao X., Zhang Z., Wang F. (2021). Recent advances in
electrocatalysis with phthalocyanines. Chem.
Soc. Rev..

[9] Ma Y., Li J., Liao X., Luo W., Huang W., Meng J., Chen Q., Xi S., Yu R., Zhao Y., Zhou L., Mai L. (2020). Heterostructure Design in Bimetallic
Phthalocyanine Boosts Oxygen Reduction Reaction Activity and Durability. Adv. Funct. Mater..

[10] Naaman R., Paltiel Y., Waldeck D. H. (2019). Chiral molecules and the electron
spin. Nature Reviews Chemistry.

[11] Tsukahara N., Noto K., Ohara M., Shiraki S., Takagi N., Takata Y., Miyawaki J., Taguchi M., Chainani A., Shin S., Kawai M. (2009). Adsorption-induced
switching of magnetic
anisotropy in a single iron­(II) phthalocyanine molecule on an oxidized
Cu(110) surface. Phys. Rev. Lett..

[12] Thole B. T., Vanderlaan G., Butler P. H. (1988). Spin-Mixed Ground-State of Fe Phthalocyanine
and the Temperature-Dependent Branching Ratio in X-Ray Absorption-Spectroscopy. Chem. Phys. Lett..

[13] Huang M., Gu Q., Wu Y., Wei Y., Pei Y., Hu T., Lutzenkirchen-Hecht D., Yuan K., Chen Y. (2025). Linkage Microenvironment
and Oxygen Electroreduction Reaction Performance Correlationship of
Iron Phthalocyanine-based Polymers. Angew. Chem.,
Int. Ed. Engl..

[14] Xie L. S., Skorupskii G., Dinca M. (2020). Electrically Conductive Metal-Organic
Frameworks. Chem. Rev..

[15] Jiang Y., Lu Y., Lv X., Han D., Zhang Q., Niu L., Chen W. (2013). Enhanced Catalytic
Performance of Pt-Free Iron Phthalocyanine by
Graphene Support for Efficient Oxygen Reduction Reaction. ACS Catal..

[16] Tang M., Zhu S., Liu Z., Jiang C., Wu Y., Li H., Wang B., Wang E., Ma J., Wang C. (2018). Tailoring
π-Conjugated Systems: From π-π Stacking to High-Rate-Performance
Organic Cathodes. Chem..

[17] Yu Y., Zhang K., Parks H., Babar M., Carr S., Craig I. M., Van Winkle M., Lyssenko A., Taniguchi T., Watanabe K., Viswanathan V., Bediako D. K. (2022). Tunable angle-dependent
electrochemistry at twisted bilayer graphene with moire flat bands. Nat. Chem..

[18] Sun Y., Sun S., Yang H., Xi S., Gracia J., Xu Z. J. (2020). Spin-Related
Electron Transfer and Orbital Interactions in Oxygen Electrocatalysis. Adv. Mater..

[19] Sun Y., Ren X., Sun S., Liu Z., Xi S., Xu Z. J. (2021). Engineering
High-Spin State Cobalt Cations in Spinel Zinc Cobalt Oxide for Spin
Channel Propagation and Active Site Enhancement in Water Oxidation. Angew. Chem., Int. Ed. Engl..

[20] Lu Y., Hu Z., Petkov P., Fu S., Qi H., Huang C., Liu Y., Huang X., Wang M., Zhang P., Kaiser U., Bonn M., Wang H. I., Samori P., Coronado E., Dong R., Feng X. (2024). Tunable Charge Transport and Spin
Dynamics in Two-Dimensional Conjugated Metal-Organic Frameworks. J. Am. Chem. Soc..

[21] Wu X., Han X., Liu Y., Liu Y., Cui Y. (2018). Control Interlayer
Stacking and Chemical Stability of Two-Dimensional Covalent Organic
Frameworks via Steric Tuning. J. Am. Chem. Soc..

[22] Zang Y., Lu D. Q., Wang K., Li B., Peng P., Lan Y. Q., Zang S. Q. (2023). A pyrolysis-free Ni/Fe bimetallic
electrocatalyst for overall water splitting. Nat. Commun..

[23] Zhang Y., Zhang X., Jiao L., Meng Z., Jiang H. L. (2023). Conductive
Covalent Organic Frameworks of Polymetallophthalocyanines as a Tunable
Platform for Electrocatalysis. J. Am. Chem.
Soc..

[24] Peng P., Shi L., Huo F., Zhang S., Mi C., Cheng Y., Xiang Z. (2019). In Situ Charge Exfoliated Soluble Covalent Organic Framework Directly
Used for Zn-Air Flow Battery. ACS Nano.

[25] Yuan C., Fu S., Kang X., Cheng C., Jiang C., Liu Y., Cui Y. (2024). Mixed-Linker Chiral 2D Covalent Organic Frameworks with Controlled
Layer Stacking for Electrochemical Asymmetric Catalysis. J. Am. Chem. Soc..

[26] Kim S. W., Jung H., Okyay M. S., Noh H. J., Chung S., Kim Y. H., Jeon J. P., Wong B. M., Cho K., Seo J. M., Yoo J. W., Baek J. B. (2023). Hexaazatriphenylene-Based
Two-Dimensional Conductive Covalent Organic Framework with Anisotropic
Charge Transfer. Angew. Chem., Int. Ed. Engl..

[27] Wang X., Zhang L., Wang N., Sun S., Wan H., Ma R., Ma W. (2024). Boosting electrocatalytic
oxygen reduction of Fe-Co
polyphthalocyanine via the synergy of metal component optimization
and axial ligand modification. Chem. Eng. J..

[28] Wei J., Xia D., Wei Y., Zhu X., Li J., Gan L. (2022). Probing the
Oxygen Reduction Reaction Intermediates and Dynamic Active Site Structures
of Molecular and Pyrolyzed Fe–N–C Electrocatalysts by
In Situ Raman Spectroscopy. ACS Catal..

[29] Wan L., Zhao K., Wang Y.-C., Wei N., Zhang P., Yuan J., Zhou Z., Sun S.-G. (2022). Molecular Degradation
of Iron Phthalocyanine during the Oxygen Reduction Reaction in Acidic
Media. ACS Catal..

[30] Kumar A., Ubaidullah M., Pandit B., Yasin G., Gupta R. K., Zhang G. (2023). Fe-phthalocyanine
derived highly conjugated 2D covalent organic framework
as superior electrocatalyst for oxygen reduction reaction. Discover. Nano.

[31] Zhan G., Koek B., Yuan Y., Liu Y., Mishra V., Lenzi V., Strutynski K., Li C., Zhang R., Zhou X., Choi H. S., Cai Z. F., Almarza J., Mali K. S., Mateo-Alonso A., Franco M. M., Zhu Y., De Feyter S., Loh K. P. (2025). Moire two-dimensional covalent organic
framework superlattices. Nat. Chem..

[32] Xu Y., Qiu F., Fu Y., Li S. F., Su X., Hong K., Zhang M. M., Zhao X., Wang Y., Xu S. Q. (2025). Solvent-Driven
Precise Control of Stacking Configurations in Covalent Organic Frameworks
for High-Efficiency Photocatalysis. Angew. Chem.,
Int. Ed. Engl..

[33] Meng F. L., Wang Z. L., Zhong H. X., Wang J., Yan J. M., Zhang X. B. (2016). Reactive Multifunctional Template-Induced Preparation
of Fe-N-Doped Mesoporous Carbon Microspheres Towards Highly Efficient
Electrocatalysts for Oxygen Reduction. Adv.
Mater..

[34] Xiao Y., Hong J., Wang X., Chen T., Hyeon T., Xu W. (2020). Revealing Kinetics of Two-Electron Oxygen Reduction Reaction at Single-Molecule
Level. J. Am. Chem. Soc..

[35] Liang Y., Li Y., Wang H., Zhou J., Wang J., Regier T., Dai H. (2011). Co­(3)­O­(4)
nanocrystals on graphene as a synergistic catalyst for
oxygen reduction reaction. Nat. Mater..

[36] Yang X., Xia D., Kang Y., Du H., Kang F., Gan L., Li J. (2020). Unveiling the Axial
Hydroxyl Ligand on Fe-N(4)-C Electrocatalysts
and Its Impact on the pH-Dependent Oxygen Reduction Activities and
Poisoning Kinetics. Adv. Sci. (Weinh).

[37] Park J. H., Lee C. H., Ju J. M., Lee J. H., Seol J., Lee S. U., Kim J. H. (2021). Bifunctional
Covalent Organic Framework-Derived
Electrocatalysts with Modulated p-Band Centers for Rechargeable Zn–Air
Batteries. Adv. Funct. Mater..

[38] Zou X., Tang M., Lu Q., Wang Y., Shao Z., An L. (2024). Carbon-based electrocatalysts for rechargeable Zn–air batteries:
design concepts, recent progress and future perspectives. Energy Environ. Sci..

[39] Lee S. H., Kim J., Chung D. Y., Yoo J. M., Lee H. S., Kim M. J., Mun B. S., Kwon S. G., Sung Y. E., Hyeon T. (2019). Design Principle
of Fe-N-C Electrocatalysts: How to Optimize Multimodal Porous Structures?. J. Am. Chem. Soc..

[40] An L., Zhang Z., Feng J., Lv F., Li Y., Wang R., Lu M., Gupta R. B., Xi P., Zhang S. (2018). Heterostructure-Promoted Oxygen Electrocatalysis Enables
Rechargeable
Zinc-Air Battery with Neutral Aqueous Electrolyte. J. Am. Chem. Soc..

[41] Yang W., Zhang L., Xie J., Zhang X., Liu Q., Yao T., Wei S., Zhang Q., Xie Y. (2016). Enhanced Photoexcited
Carrier Separation in Oxygen-Doped ZnIn2 S4 Nanosheets for Hydrogen
Evolution.. Angew. Chem., Int. Ed. Engl..

[42] Li Z., Deng T., Ma S., Zhang Z., Wu G., Wang J., Li Q., Xia H., Yang S. W., Liu X. (2023). Three-Component Donor-pi-Acceptor
Covalent-Organic Frameworks for
Boosting Photocatalytic Hydrogen Evolution.. J. Am. Chem. Soc..

[43] Vighnesh K., Sergeev A. A., Hassan M. S., Portniagin A. S., Sokolova A. V., Zhu D., Sergeeva K. A., Kershaw S. V., Wong K. S., Rogach A. L. (2024). Red-Emitting CsPbI(3)/ZnSe
Colloidal
Nanoheterostructures with Enhanced Optical Properties and Stability.. Small.

[44] Hassan M. S., Basera P., Khan B., Portniagin A. S., Vighnesh K., Wu Y., Rusanov D. A., Babak M., He J. H., Bajdich M., Rogach A. L. (2025). Bidentate Lewis
Base Ligand-Mediated Surface Stabilization and Modulation of the Electronic
Structure of CsPbBr(3) Perovskite Nanocrystals.. J. Am. Chem. Soc..

[45] Lyu D., Mollamahale Y. B., Huang S., Zhu P., Zhang X., Du Y., Wang S., Qing M., Tian Z. Q., Shen P. K. (2018). Ultra-high
surface area graphitic Fe-N-C nanospheres with single-atom iron sites
as highly efficient non-precious metal bifunctional catalysts towards
oxygen redox reactions.. J. Catal..

[46] Li L., Tang X., Huang S., Lu C., Lutzenkirchen-Hecht D., Yuan K., Zhuang X., Chen Y. (2023). Longitudinally Grafting
of Graphene with Iron Phthalocyanine-based Porous Organic Polymer
to Boost Oxygen Electroreduction.. Angew. Chem.,
Int. Ed. Engl..

[47] Mei B., Mao J., Liang Z., Sun F., Yang S., Li J., Ma J., Song F., Jiang Z. (2025). Reversible Angle Distortion-Dependent
Electrochemical CO(2) Reduction on Cobalt Phthalocyanine.. J. Am. Chem. Soc..

[48] Chang Q., Liu Y., Lee J. H., Ologunagba D., Hwang S., Xie Z., Kattel S., Lee J. H., Chen J. G. (2022). Metal-Coordinated
Phthalocyanines as Platform Molecules for Understanding Isolated Metal
Sites in the Electrochemical Reduction of CO(2).. J. Am. Chem. Soc..

[49] Zhuang Z., Xia L., Huang J., Zhu P., Li Y., Ye C., Xia M., Yu R., Lang Z., Zhu J., Zheng L., Wang Y., Zhai T., Zhao Y., Wei S., Li J., Wang D., Li Y. (2023). Continuous Modulation of Electrocatalytic
Oxygen Reduction Activities of Single-Atom Catalysts through p-n Junction
Rectification. Angew. Chem., Int. Ed. Engl..

[50] Chi H., Liu Y., Li Z., Chen W., He Y. (2023). Direct visual observation
of pedal motion-dependent flexibility of single covalent organic frameworks. Nat. Commun..

[51] Zhang S., Han Y., Zhang R., Zhang Z., Sun G. (2024). Regulating Fe Intermediate
Spin States via FeN4-Cl-Ti Structure for Enhanced Oxygen Reduction. Adv. Energy Mater..

[52] Yang G., Zhu J., Yuan P., Hu Y., Qu G., Lu B. A., Xue X., Yin H., Cheng W., Cheng J., Xu W., Li J., Hu J., Mu S., Zhang J. N. (2021). Regulating Fe-spin
state by atomically dispersed Mn-N in Fe-N-C catalysts with high oxygen
reduction activity. Nat. Commun..

[53] Zhu Y., Jiang Y., Li H., Zhang D., Tao L., Fu X. Z., Liu M., Wang S. (2024). Tip-like Fe-N(4) Sites
Induced Surface Microenvironments Regulation Boosts the Oxygen Reduction
Reaction. Angew. Chem., Int. Ed. Engl..

[54] Wang X., Yi Z. Y., Wang Y. Q., Wang D. (2025). Molecular Evidence
for the Axial Coordination Effect of Atomic Iodine on Fe-N(4) Sites
in Oxygen Reduction Reaction. Angew. Chem.,
Int. Ed. Engl..

[55] Fernández-Rodríguez J., Toby B., van Veenendaal M. (2015). Mixed configuration ground state
in iron­(II) phthalocyanine. Phys. Rev. B.

[56] Zhang X., Wolf C., Wang Y., Aubin H., Bilgeri T., Willke P., Heinrich A. J., Choi T. (2022). Electron spin resonance
of single iron phthalocyanine molecules and role of their non-localized
spins in magnetic interactions. Nat. Chem..

[57] Liu K., Fu J., Lin Y., Luo T., Ni G., Li H., Lin Z., Liu M. (2022). Insights into the activity of single-atom Fe-N-C catalysts
for oxygen reduction reaction. Nat. Commun..

[58] Qiao J., Lu C., Kong L., Zhang J., Lin Q., Huang H., Li C., He W., Zhou M., Sun Z. (2024). Spin Engineering of
Fe—N—C by Axial Ligand Modulation for Enhanced Bifunctional
Oxygen Catalysis. Adv. Funct. Mater..

[59] Wang R., Zhang L., Shan J., Yang Y., Lee J. F., Chen T. Y., Mao J., Zhao Y., Yang L., Hu Z., Ling T. (2022). Tuning Fe
Spin Moment in Fe-N-C Catalysts to Climb
the Activity Volcano via a Local Geometric Distortion Strategy. Adv. Sci. (Weinh).

[60] Zheng T., Wang J., Xia Z., Wang G., Duan Z. (2023). Spin-dependent
active centers in Fe–N–C oxygen reduction catalysts
revealed by constant-potential density functional theory. Journal of Materials Chemistry A.

[61] Zhu Y., Prezhdo O. V., Long R., Fang W. H. (2023). Twist Angle-Dependent
Intervalley Charge Carrier Transfer and Recombination in Bilayer WS(2). J. Am. Chem. Soc..

[62] Grimme S. (2011). Density functional
theory with London dispersion corrections. WIREs
Computational Molecular Science.

[63] Goerigk L., Grimme S. (2011). A thorough benchmark
of density functional methods
for general main group thermochemistry, kinetics, and noncovalent
interactions. Phys. Chem. Chem. Phys..

