# Supplementary material for: Stacking Order-Mediated Spin-State Modulation in Iron Phthalocyanine Covalent Organic Frameworks Enables Efficient Oxygen Reduction Reaction
Source: ACS Appl Mater Interfaces. 2026 Jan 23;18(4):6735–46. doi: 10.1021/acsami.5c19809 (PMC12884466; doi:10.1021/acsami.5c19809)
Supplement: Supplementary file 1 [file am5c19809_si_001.pdf]

Supporting Information

Stacking Order Mediated Spin State Modulation in Iron-Phthalocyanine Covalent Organic Frameworks Enables Efficient Oxygen Reduction Reaction

Yun Li,[a] *Md. Samim Hassan,*[a] *Desui Chen,*[a] *Yuxuan Wu,*[b] *Xin Zhao,*[c] *Arsenii S. Portniagin,*[a] *Haochen Liu,*[a] *Shixun Wang,*[a] *Penghui Ren,*[d] *Ying Zhao,*[d] *Andrey L. Rogach*[*a]

[a] Department of Materials Science and Engineering, City University of Hong Kong, 83 Tat Chee Avenue, Kowloon, Hong Kong S.A.R. 999077, P. R. China

[b] Department of Chemistry, City University of Hong Kong, 83 Tat Chee Avenue, Kowloon, Hong Kong S.A.R. 999077, P. R. China

[c] Department of Materials Science and Engineering, Hainan University, 58 Renmin Avenue, Haikou 570000, P. R. China

[d] Shandong Laboratory of Advanced Materials and Green Manufacturing, Yantai 264000, P. R. China

[*]E-mail: andrey.rogach@cityu.edu.hk

Experimental Section

Calculation of the Valence Band Center and the Fermi Level. The Shirley background was first subtracted from the measured valence band spectrum radiated by the He I source with an energy of 21.2 eV. The position of the valence band center is given by: $\int N(\varepsilon)\varepsilon d\varepsilon / \int N(\varepsilon) d\varepsilon$, where $N(\varepsilon)$ is the measured XPS-intensity after background subtraction. The background calibrated spectra were integrated from the binding energy 10 eV up to the Fermi level value.[1-4] The Fermi level was measured by subtracting the He I radiation energy of 21.2 eV from the high-binding energy cut-off in the ultraviolet photoelectron spectra.

Calculation of the Magnetic Effective Moment and the Number of Unpaired Electrons. The Curie-Weiss law indicates that the magnetic susceptibility (χ) is inversely proportional to the temperature:

$$\chi = \frac{C}{T} = \frac{N^2 g^2 \beta^2 \mu_B S(S+1)}{3kT}$$

where $\frac{N^2\beta^2}{3k}$ is a constant equal to 0.125, μ_B is the Bohr magneton, S is the spin state, and g is the Landé g factor. The magnetic effective moment (μ_{eff}) can be calculated as:[5]

$$\mu_{eff} = \sqrt{g^2 S(S+1)}\mu_B$$

Thus, μ_{eff} can be derived from χ via the following equation:

$$\mu_{eff} = \sqrt{8\chi T}\mu_B$$

The number of unpaired electrons (n) is associated with μ_{eff}, which can be calculated from the equation:[6-8]

$$\mu_{eff} = \sqrt{n(n+1)}\mu_B$$

Computational Details. Density Functional Theory (DFT) calculations were performed by the Vienna *ab-initio* Simulation Package (VASP) using Perdew-Burke-Ernzerh (PBE) method, where the Grimme DFT-D3(BJ) module was used to correct the weak van der Waals interaction term.[9] Becke-Johnson damping function was chosen to describe intramolecular dispersion. We used the plane wave basis with a cut-off energy of 500 eV, electronic self-consistent field 1×10^{-6} eV, ionic convergence energy 0.02 eV Å^{-1}, and Gaussian electron smearing width 0.05 eV for geometry optimization,

respectively. Spin-polarized calculations were performed to determine the spin density of FePc COFs, which can be calculated by:

$$\rho^{spin} = |\rho^{up} - \rho^{down}|$$

where ρ^{up} and ρ^{down} represent the electron density with up and down spin states, respectively. The charge difference density between A and B parts was calculated as:

$$\Delta\rho = \rho^{AB} - \rho^{A} - \rho^{B}$$

where ρ^{AB}, ρ^{A}, and ρ^{B} denote the charge density of the systems AB, A and B. We further proceeded the Gibbs free energies for the oxygenate intermediates involved in the ORR process. The ORR mechanism was determined to follow the 4-electron transfer pathway by detecting the yield ratio of hydrogen peroxide on a rotating ring-disk electrode:

$$O_2(g) + * \rightarrow *O_2 \qquad \Delta G1$$

$$*O_2 + H_2O + e^- \rightarrow *OOH + OH^- \qquad \Delta G2$$

$$*OOH + e^- \rightarrow *O + OH^- \qquad \Delta G3$$

$$*O + H_2O + e^- \rightarrow *OH + OH^- \qquad \Delta G4$$

$$*OH + e^- \rightarrow * + OH^- \qquad \Delta G5$$

Overall reaction:

$$O_2(g) + H_2O\ (l) + 4\,e^- \rightarrow 4\,OH^- \qquad \Delta G = -\,4.92\ eV$$

The Gibbs free energy of each elementary step was calculated as:

$$\Delta G = \Delta E + \Delta ZPE - T\Delta S$$

where ΔE represents the reaction energy, while ΔZPE and $T\Delta S$ represent two correction terms – zero-point energy and entropy. ΔE can be calculated by the subduction of total electronic energy of products and reactants, and ΔZPE and $T\Delta S$ can be derived from the vibration frequency of the phonons.

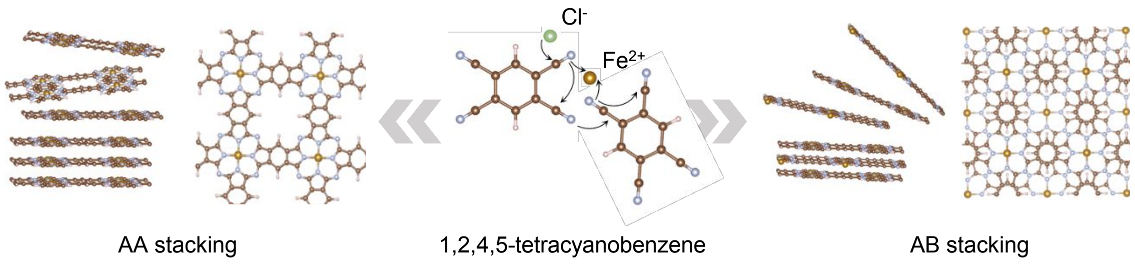

Figure S1. Schematic illustration of the synthesis of eclipsed (AA-stacked) and staggered (AB-stacked) FePc COFs.

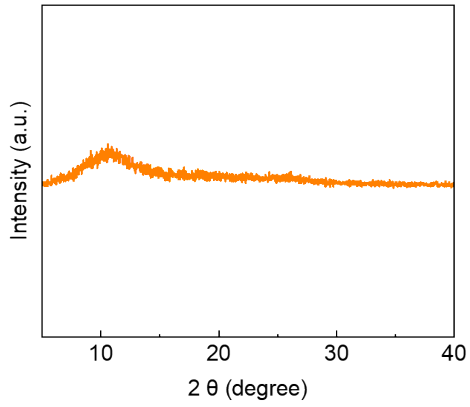

Figure S2. Powder XRD pattern of COF-Fe-35.

Table S1. Pawley and Rietveld refined lattice parameters for COF-Fe-75 (AA stacking) and COF-Fe-150 (AB stacking).

cell		lengths (Å)			angles (°)			agreement factors (%)	
		a	b	C	alpha	beta	gamma	R_{wp}	R_p
Pawley	AA	9.8 ± 0.001	9.5 ± 0.001	3.0 ± 0.001	90	90	90	3.5	2.6
	AB	10.8 ± 0.001	10.6 ± 0.001	6.4 ± 0.001	90	90	90	4.2	2.0
Rietveld	AA	9.8 ± 0.001	9.5 ± 0.001	3.0 ± 0.001	90	90	90	5.0	1.5
	AB	10.8 ± 0.003	10.7 ± 0.003	6.5 ± 0.001	90	90	90	6.3	2.5

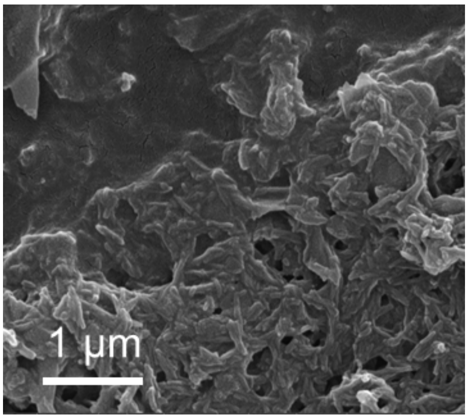

Figure S3. SEM image of COF-Fe-35.

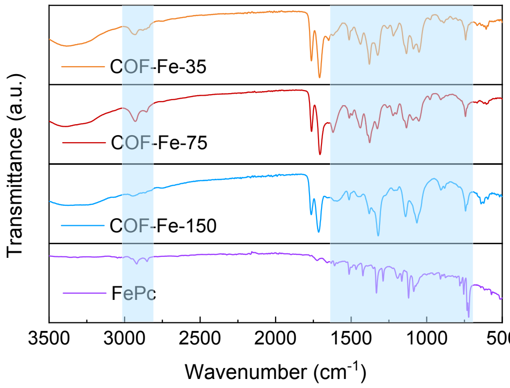

Figure S4. FTIR spectra of COF-Fe-35, COF-Fe-75, COF-Fe-150 and FePc.

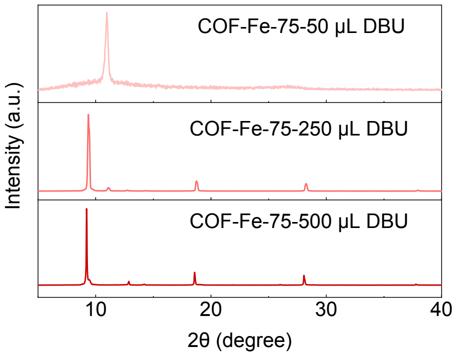

Figure S5. Powder XRD patterns of COF-Fe-75-50 μL DBU, COF-Fe-75-250 μL DBU, and COF-Fe-75-500 μL DBU. The three samples were synthesized using fixed amount of $FeCl_2$ (75 mg) with varying the amount of DBU (50 μL, 250 μL, and 500 μL, as included in the sample's name), whereas the temperature and synthesis time were fixed at 180 °C and 24 h, respectively.

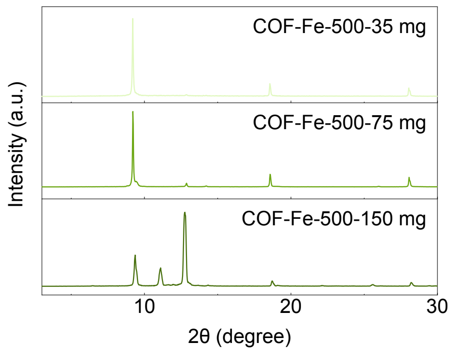

Figure S6. Powder XRD patterns of COF-Fe-500-35 mg, COF-Fe-500-75 mg, and COF-Fe-500-150 mg. The three samples were synthesized using fixed amount of DBU (500 μL) with varying the amount of $FeCl_2$ (35 mg, 75 mg, and 150 mg, as included in the sample's name), whereas the temperature and synthesis time were set at 180 °C and 24 h, respectively.

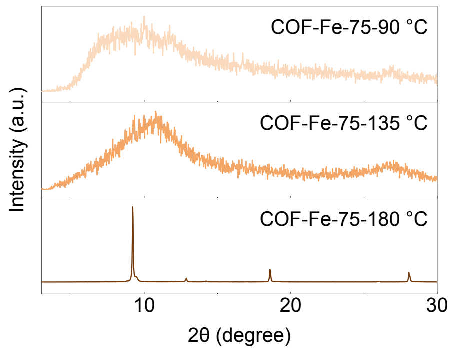

Figure S7. Powder XRD patterns of COF-Fe-75-90 ℃, COF-Fe-75-135 ℃, and COF-Fe-75-180 ℃. These samples were synthesized using fixed amount of 75 mg of $FeCl_2$ and 500 μL of DBU under different reaction temperature of 90 ℃, 135 ℃, and 180 ℃.

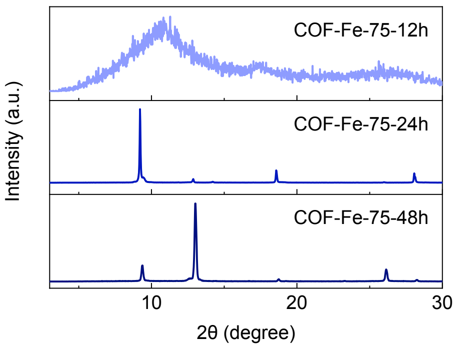

Figure S8. Powder XRD patterns of COF-Fe-75-12h, COF-Fe-75-24h, and COF-Fe-75-48h. These samples were synthesized using fixed amount of 75 mg of $FeCl_2$ and 500 μL of DBU under different reaction time of 12 h, 24 h, and 48 h.

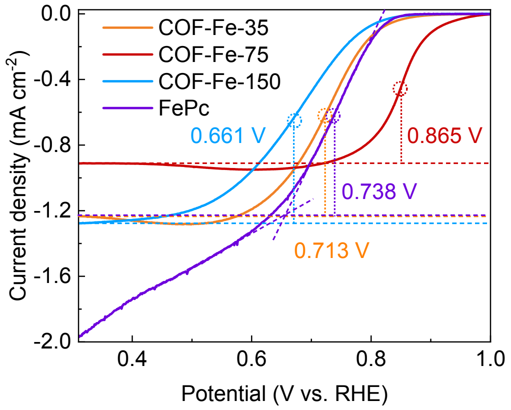

Figure S9. Determination of half-wave potential from the LSV curves for COF-Fe-35, COF-Fe-75, COF-Fe-150, and FePc.

The half-wave potential was determined as the specific potential at a current density with half of the limited current density in LSV curves. Apart from FePc, the limitation current densities for all samples are shown as the horizontal dash curves of specific colors. The limited current density for FePc was determined as the intersection point of two tangent plots of its LSV curve.

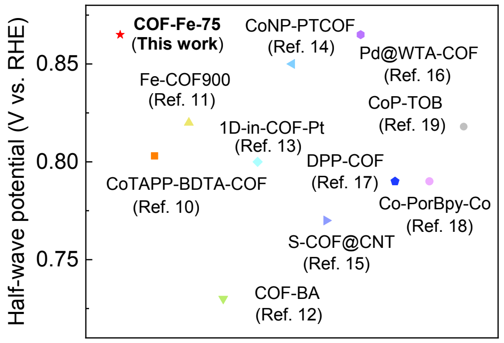

Figure S10. Comparison of the ORR activity (in 0.1 M KOH) of COF-Fe-75 synthesized here (red star) with other reported metal coordinated COFs electrocatalysts.[10-19]

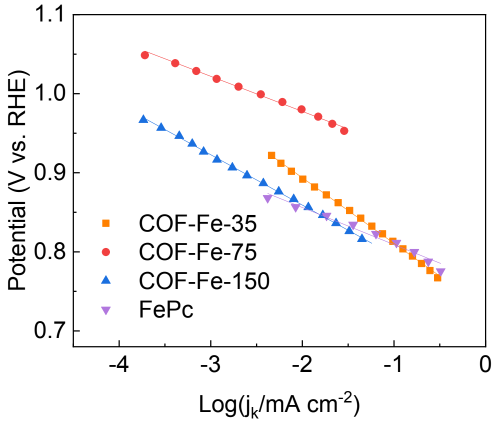

Figure S11. Tafel plots derived from ORR polarization curves for COF-Fe-35, COF-Fe-75, COF-Fe-150 and FePc.

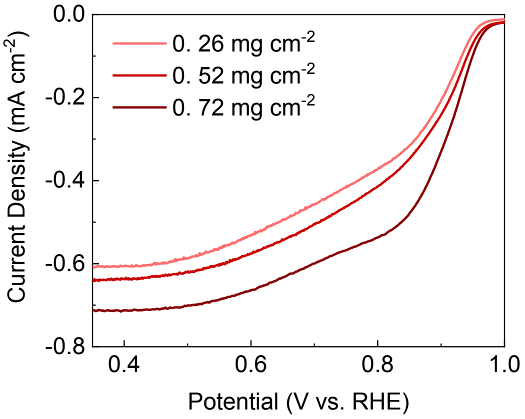

Figure S12. LSV curves of COF-Fe-75 with different loading of 0.26 mg cm^{-2}, 0.52 mg cm^{-2}, and 0.72 mg cm^{-2}.

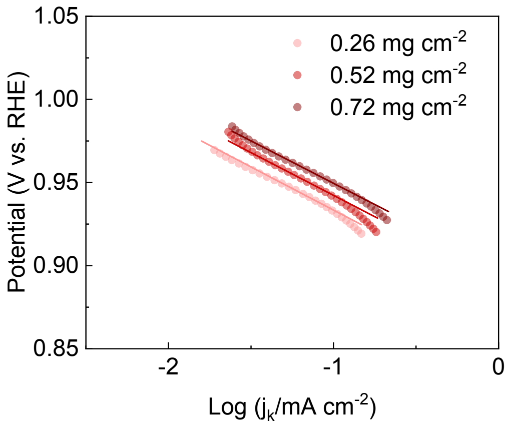

Figure S13. Tafel plots derived from ORR polarization curves for COF-Fe-75 with

different loading (0.26 mg cm^{-2}, 0.52 mg cm^{-2}, and 0.72 mg cm^{-2}).

Tafel slopes for different catalyst loadings of 0.26 mg cm^{-2}, 0.52 mg cm^{-2}, and 0.72 mg cm^{-2} are 51.9 mV dec^{-1}, 51.4 mV dec^{-1}, and 51.0 mV dec^{-1}, respectively. The nearly constant Tafel slopes indicates the same ORR mechanism catalyzed by COF-Fe-75, even as the loading increases.

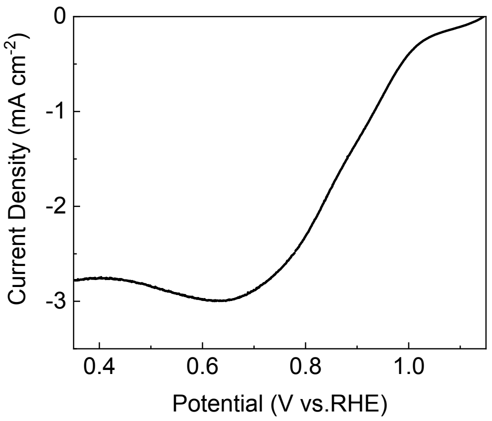

Figure S14. LSV curve of 20% Pt/C with the loading of 0.50 mg cm^{-2}, the half-wave potential of which is 0.894 V vs. RHE.

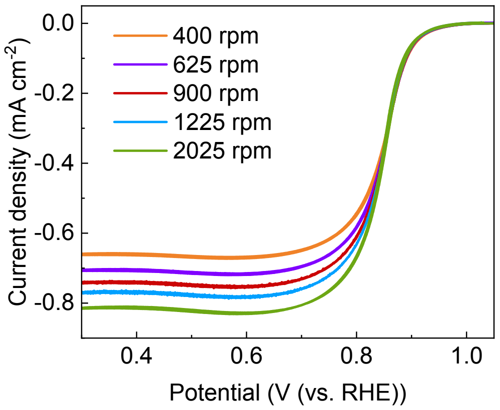

Figure S15. LSV curves of COF-Fe-75 measured at different rotation speeds of 400 rpm, 625 rpm, 900 rpm, 1225 rpm, and 2025 rpm.

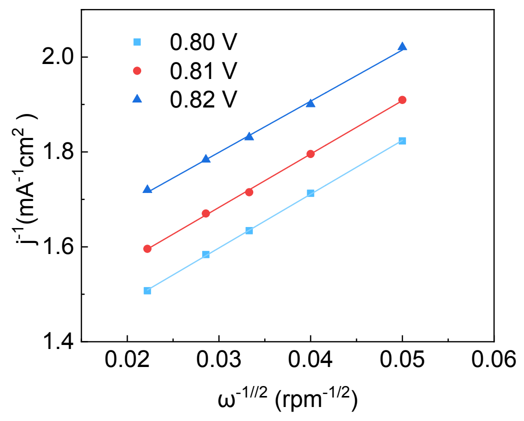

Figure S16. Koutecky-Levich plots of COF-Fe-75 derived from the LSV curves measured with varying rotation speed, where the estimated electron transfer number is 4.005.

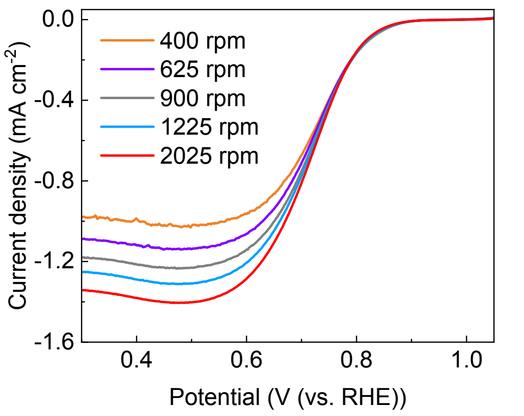

Figure S17. LSV curves of COF-Fe-35 measured at different rotation speeds of 400 rpm, 625 rpm, 900 rpm, 1225 rpm, and 2025 rpm.

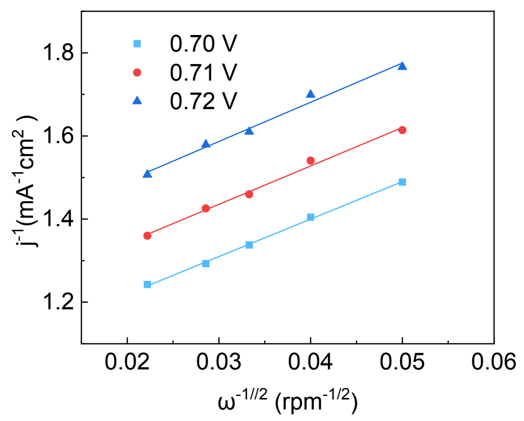

Figure S18. Koutecky-Levich plots of COF-Fe-35 derived from the LSV curves with

varying rotation speed, where the estimated electron transfer number is 3.402.

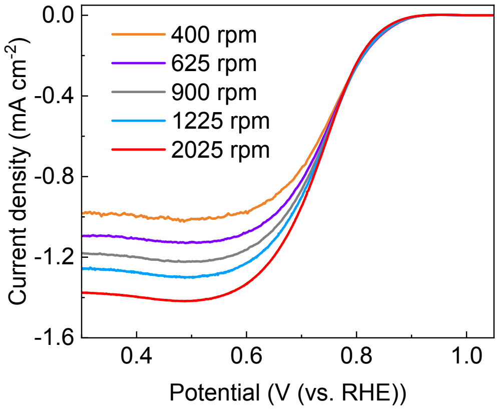

Figure S19. LSV curves of COF-Fe-150 measured at different rotation speeds of 400 rpm, 625 rpm, 900 rpm, 1225 rpm, and 2025 rpm.

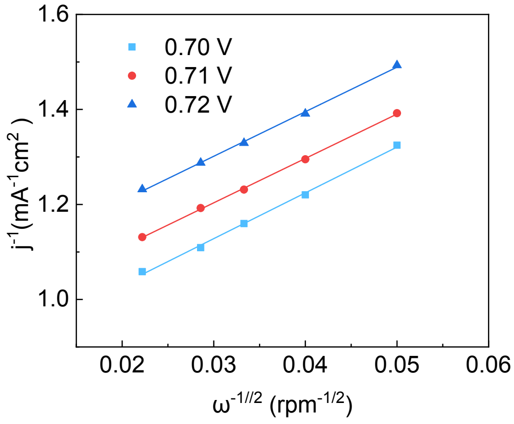

Figure S20. Koutecky-Levich plots of COF-Fe-150 derived from the LSV curves with varying rotation speed, where the estimated electron transfer number is 3.322.

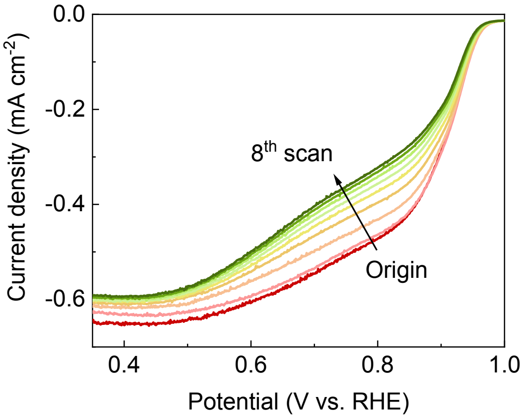

Figure S21. LSV curves of COF-Fe-75 measured under continuous 8 scans after adding

0.1 mM KSCN into 0.1 M KOH electrolyte.

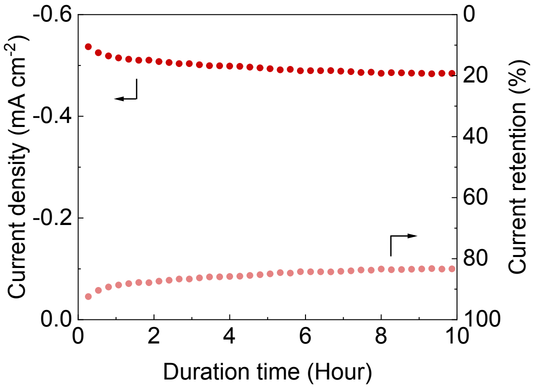

Figure S22. Chronoamperometric response of COF-Fe-75 for continuous ORR at 0.6 V vs. RHE.

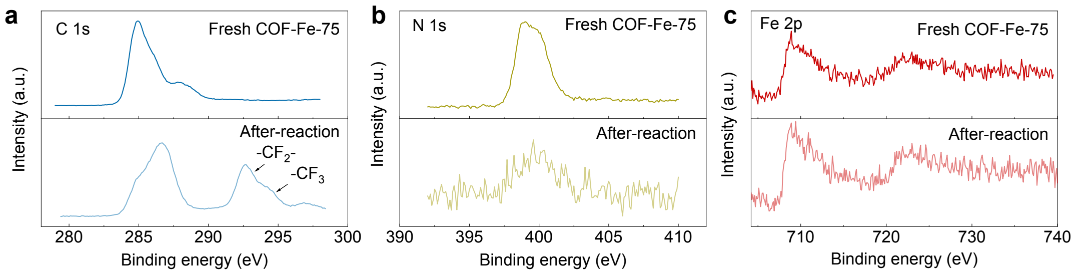

Figure S23. High-resolution XPS spectra of (a) C 1s, (b) N 1s, and (c) Fe 2p peaks for COF-Fe-75 before and after 10-h operation at 0.6 V vs. RHE in a RRDE system.

Strong characteristic peaks of -CF_2- and -CF_3 for Nafion 117 appear at 292.7 eV and 294.5 eV in COF-Fe-75 (**Figure S23a**), which significantly influence the analysis of the actual carbon state in this sample. Regarding the N 1s spectra, there is no noticeable change after continuous operation. Additionally, the Fe 2p characteristic peaks demonstrate that the oxidation state of iron in COF-Fe-75 remains the same after a 10-h duration measurement.

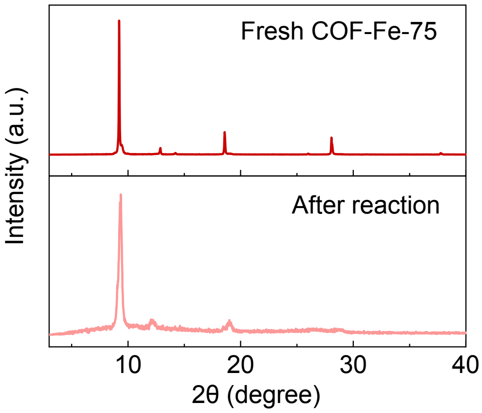

Figure S24. Powder XRD pattern of COF-Fe-75 before and after 10-h operation at 0.6 V vs. RHE shows almost no change in the lattice structure compared to the fresh COF-Fe-75 sample. This indicates that the material has outstanding structure stability.

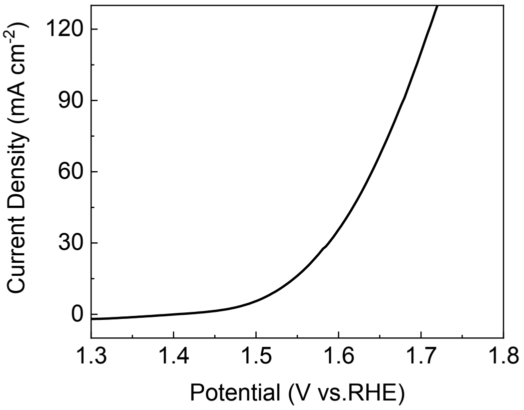

Figure S25. LSV curve of commercial RuO_2 in the OER process, the overpotential of which is 296 mV at 10 mA cm^{-2}.

Table S2. Comparison of bifunctional COF electrocatalysts (ORR and OER performance) with COF-Fe-75 reported in this work.

COFs	ORR (V vs. RHE)	OER (mV)	electrolyte	reference
CoNP-PTCOF	0.85	450	0.1 M KOH	14
CoTAPP-PATA-COF	0.80	420	0.1 M KOH	10
CoP-TOB	0.818	450	0.1 M KOH	19
CC-3	0.828	389	0.1 M KOH	22
CoNP-s-IMCOF	0.83	500	0.1 M KOH	23

SUZ-101-Co	0.78	240	0.1 M KOH	24
Ag/Ag_2O@MCOF(Co)	0.76	260 (1 M KOH)	0.1 M KOH	25
$CoFe_2O_4$@CN/CTF-2	0.84	330	1.0 M KOH	26
MCAC	0.8	220	0.1 M KOH	27
A-PpazoPorCo	0.88	298	1.0 M KOH	28
COF-Fe-75	0.856	281 (1 M KOH)	0.1 M KOH	**This work**

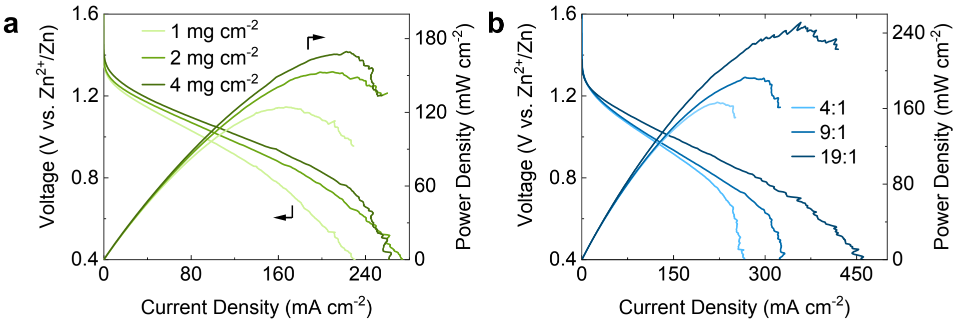

Figure S26. Polarization and power density curves of zinc-air batteries using COF-Fe-75 as cathodes with (a) different loading and (b) different amount of Nafion 117.

Here, we systematically examined the effects of different loading levels of electrocatalysts and varying amounts of Nafion 117 as a binder on the power density of zinc-air batteries assembled using COF-Fe-75 as the cathode. As illustrated in **Figure S26a**, the power density gradually increases from 124.1 to 169.3 mW cm^{-2} with a four-fold increase in catalyst loading. The addition of Nafion 117 has a significant influence on the power density of battery: we observed an increase of 84.6 mW cm^{-2} when a smaller amount of Nafion 117 was utilized, specifically when the volume ratio of methanol to Nafion increased from 4:1 to 19:1(**Figure S26b**).

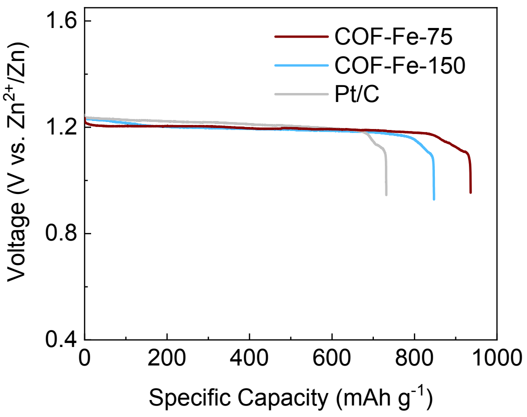

Figure S27. Specific capacities of the assembled zinc-air batteries measured at 10 mA cm^{-2} using COF-Fe-75, COF-Fe-150, and 20% Pt/C as cathodes.

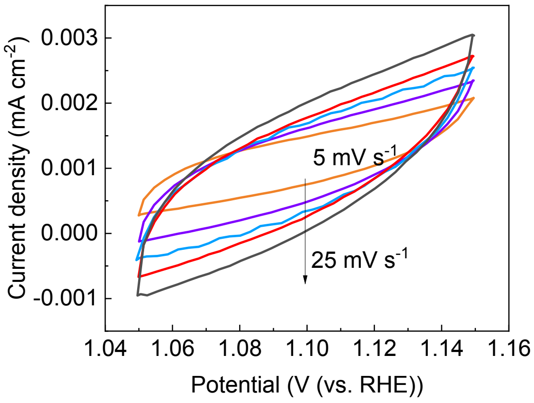

Figure S28. CV curves of COF-Fe-75 measured at 5 mV s^{-1}, 10 mV s^{-1}, 15 mV s^{-1}, 20 mV s^{-1}, and 25 mV s^{-1}.

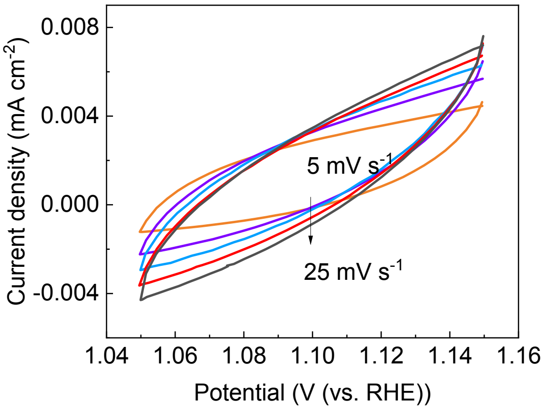

Figure S29. CV curves of COF-Fe-35 measured at 5 mV s^{-1}, 10 mV s^{-1}, 15 mV s^{-1}, 20 mV s^{-1}, and 25 mV s^{-1}.

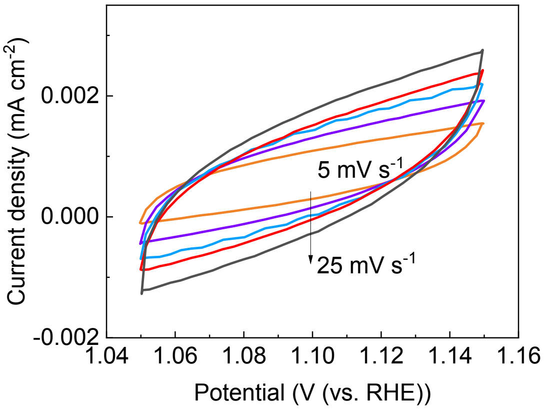

Figure S30. CV curves of COF-Fe-150 measured at 5 mV s^{-1}, 10 mV s^{-1}, 15 mV s^{-1}, 20 mV s^{-1}, and 25 mV s^{-1}.

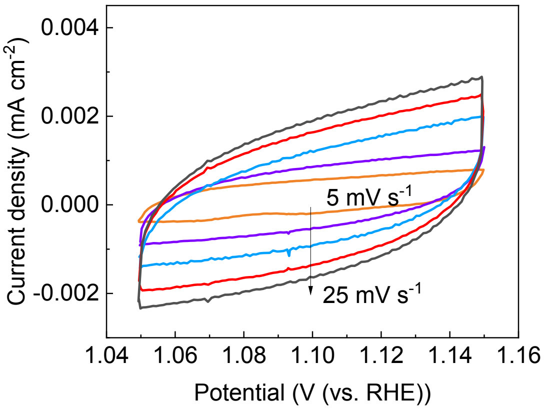

Figure S31. CV curves of FePc measured at 5 mV s^{-1}, 10 mV s^{-1}, 15 mV s^{-1}, 20 mV s^{-1}, and 25 mV s^{-1}.

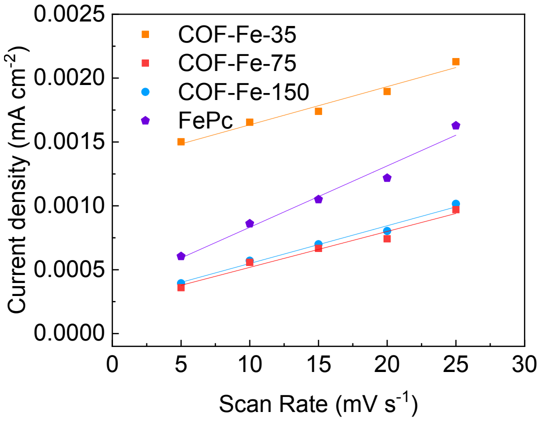

Figure S32. Plots of current densities (at 1.10 V vs. RHE) as a function of scan rates for COF-Fe-35, COF-Fe-75, COF-Fe-150, and FePc.

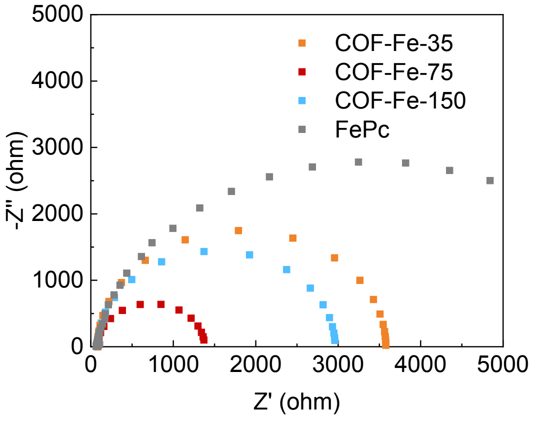

Figure S33. Nyquist plots of COF-Fe-35, COF-Fe-75, COF-Fe-150, and FePc.

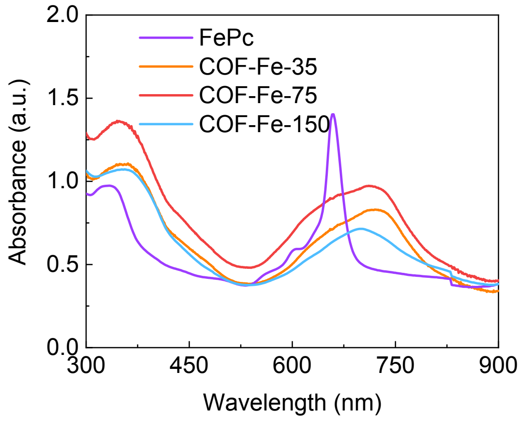

Figure S34. Absorption spectra of COF-Fe-35, COF-Fe-75, COF-Fe-150, and FePc.

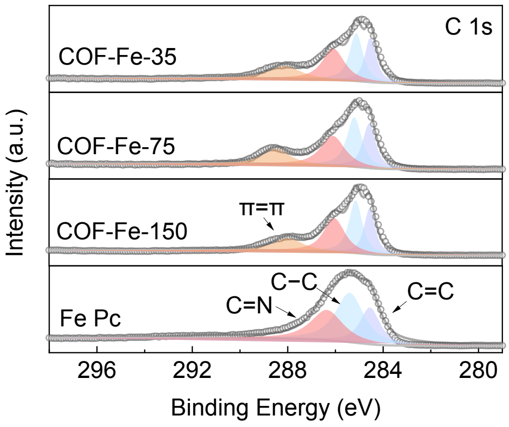

Figure S35. High-resolution XPS spectra for C 1s peaks in COF-Fe-35, COF-Fe-75, COF-Fe-150, and FePc.

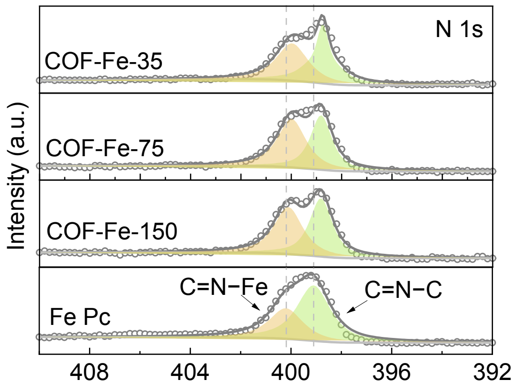

Figure S36. High-resolution XPS spectra for N 1s peaks in COF-Fe-35, COF-Fe-75, COF-Fe-150, and FePc.

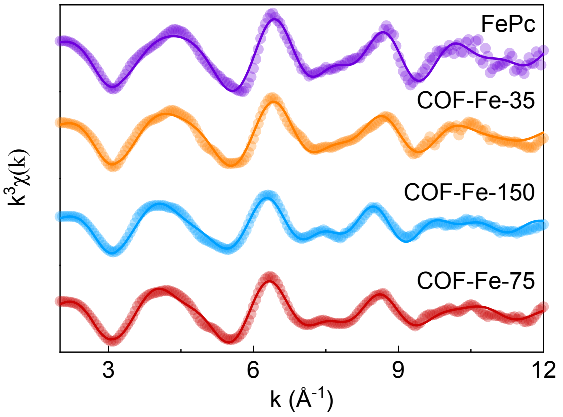

Figure S37. Re($k^3\chi(k)$) oscillations of FePc, COF-Fe-35, COF-Fe-75, and COF-Fe-150 of the k^3-weighted Fe K-edge EXAFS.

Table S3. Fe K-edge EXAFS analysis for the FePc, COF-Fe-150, and COF-Fe-75

	path	CN	R (Å)	σ^2 (Å^2)	ΔE_0 (eV)	R-factor
FePc	Fe-N	4.28	1.97±0.012	0.00630	1.207	0.0196
COF-Fe-35	Fe-N	4.20	1.98±0.015	0.00798	-0.142	0.0128
COF-Fe-150	Fe-N	4.50	2.01±0.014	0.00880	0.630	0.0126
COF-Fe-75	Fe-N	4.56	1.95±0.015	0.00794	-7.730	0.0130

Note: CN, coordination number; R, distance between absorber and backscatter atoms;

σ^2, Debye–Waller factor to account for both thermal and structural disorders; ΔE_0 (eV), inner potential correction to account for the difference in the inner potential between the sample and the reference compound; R-factor indicates the goodness of the fit. The amplitude reduction factor (S_0^2) was set to 1.0 during the fitting process.

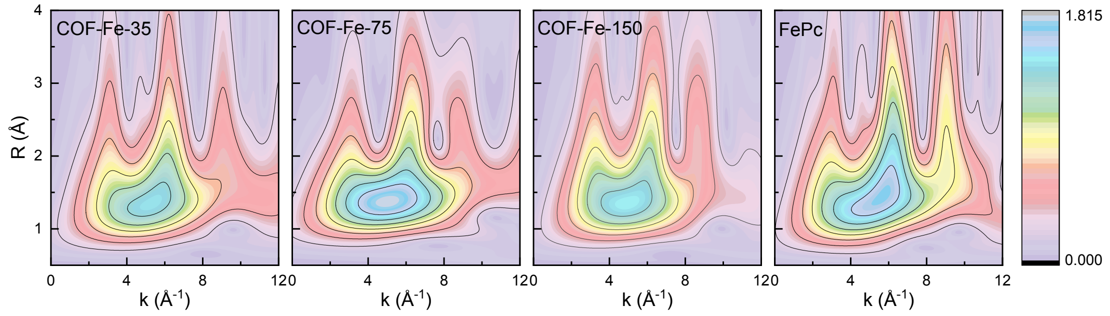

Figure S38. Wavelet transform contour plots of COF-Fe-35, COF-Fe-75, COF-Fe-150, and FePc.

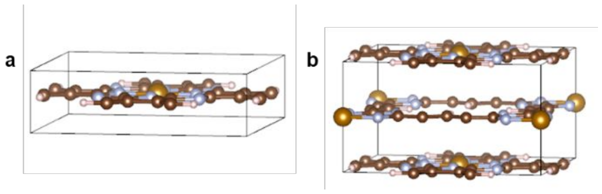

Figure S39. Unit cells of (a) eclipsed, AA-stacked FePc COF and (b) staggered, AB-stacked FePc COF.

Table S4. Simulated lattice parameters of eclipsed, AA-stacked FePc COF and staggered, AB-stacked FePc COF.

lattice parameters	a (Å)	b (Å)	c (Å)	alpha (°)	beta (°)	gamma (°)	space group
AA stacking	10.3	10.3	3.0	90	90	90	P4/mmm
AB stacking	10.4	10.4	5.7	90	90	90	I4/mmm

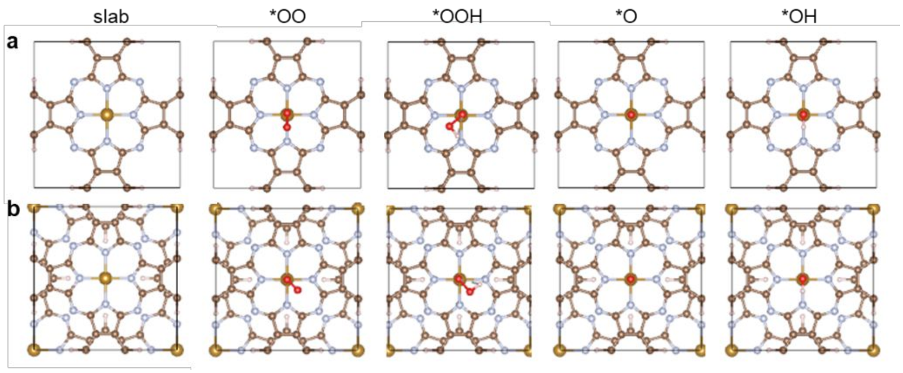

Figure S40. The 4-electron transfer mechanism of ORR at the Fe-N_4 moiety within the (0 0 1) plane of the (a) AA-stacked and (b) AB-stacked FePc COFs.

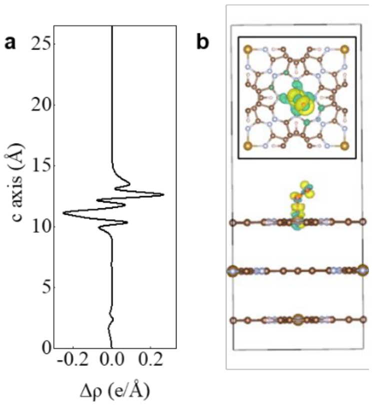

Figure S41. (a) Average charge density difference as a function of the position along the *c* axis, and (b) 3D charge density difference in AB-stacked FePc COF, where the yellow and cyan regions denote the depletion and accumulation of the electrons, respectively.

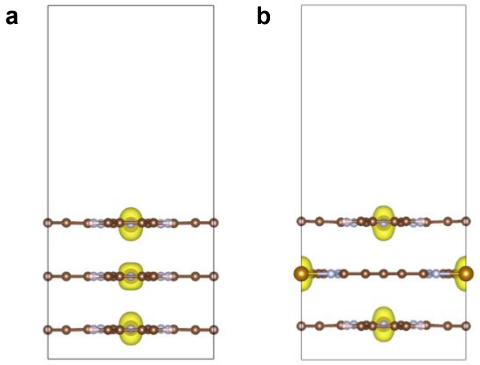

Figure 42. The distribution of spin density in (a) AA-stacked and (b) AB-stacked FePc COFs, where the yellow and cyan regions represent the distribution of spin density with opposite spin orientation.

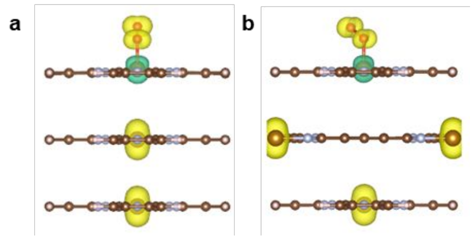

Figure S43. Spin density of (a) AA-stacked and (b) AB-stacked FePc COF when adsorbing an oxygen molecule at Fe site, where the yellow and cyan regions represent the distribution of spin density with opposite spin orientation.

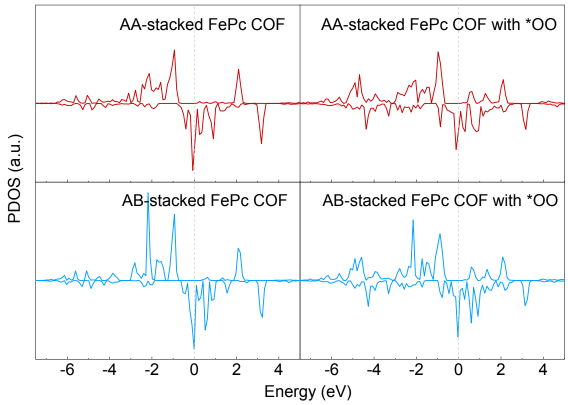

Figure S44. Projected DOS (PDOS) for the *d* orbitals of Fe in AA-stacked and AB-stacked FePc COFs (three monolayers) with and without *OO adsorbates.

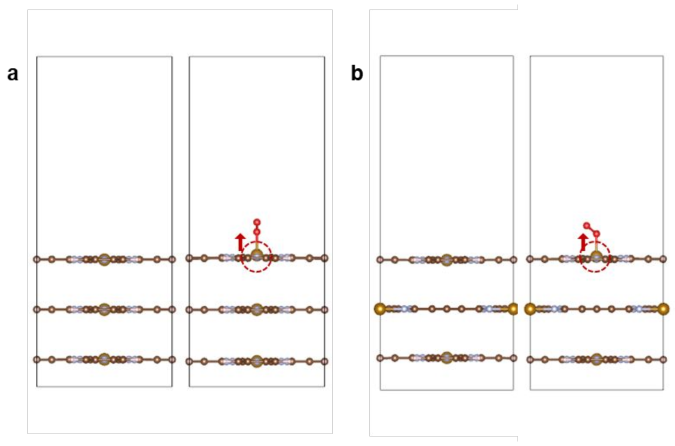

Figure S45. The front view of (a) AA-stacked and (b) AB-stacked FePc COFs without (left) and with (right) adsorbed of oxygen molecule.

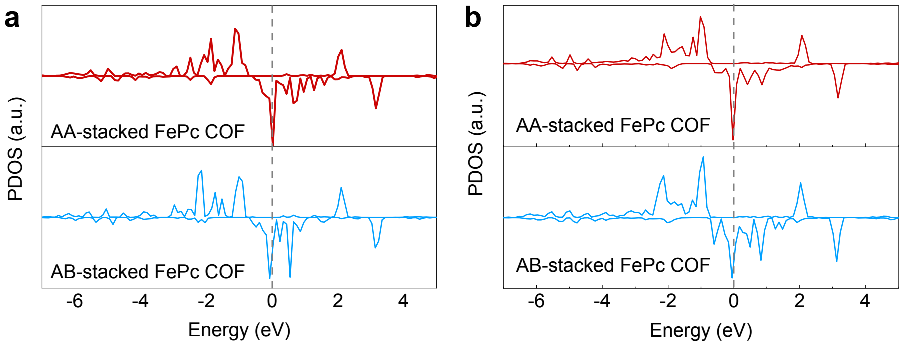

Figure S46. PDOS for the *d* orbitals of Fe in (a) bulk and (b) double-layered FePc COFs with an AA-stacking and AB-stacking configuration.

In the case of the same number of stacking layers, the *d*-band center of Fe in the AA-stacked FePc COF is closer to the Fermi level compared to AB-stacked one. For the bulk AA-stacked FePc COFs, the *d*-band center is at -1.68 eV, which is higher than that for the bulk in AB-stacking mode (-1.91 eV). We also found that the *d*-band center of Fe shifts towards the Fermi level with increasing number of stacking layers, evidenced by the lower *d*-band center of double-layered AA-stacked FePc COF (-1.86 eV) compared with the bulk one.

The Fe site in the AA-stacked FePc COF exhibits a higher spin state compared to that in AB-stacked configuration, regardless of the number of stacking layers. Specifically, the spin states (defined as the difference between the integrating up and down spin state below Fermi level) in the AA-stacked structure are 0.59 and 0.32 higher than that in AB-stacked arrangement for both the double layered and bulk FePc COF model, respectively. At the same time, we observed that the spin of Fe in the bulk FePc COF is stronger than in the double-layered FePc COF. Overall, the FePc COF in the AA-stacking mode consistently shows an enhanced spin state compared to that in the AB-stacking mode.

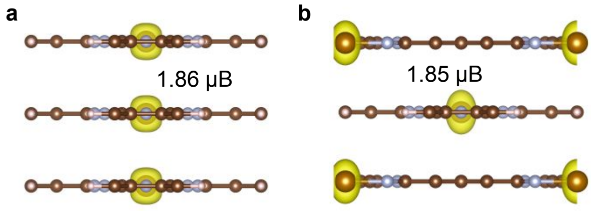

Figure S47. Spin distribution and atomic magnetic moment of Fe sites in (a) AA-stacked FePc COF and (b) AB-stacked FePc COF. The atomic magnetic moment of Fe site in the AA-stacked FePc COF is 1.86 μ_B, which is higher than that of AB-stacked

FePc COF (1.85 μ_B).

REFERENCES

1. Wu, X.; Chen, F.; Zhang, N.; Lei, Y.; Jin, Y.; Qaseem, A.; Johnston, R. L., Activity Trends of Binary Silver Alloy Nanocatalysts for Oxygen Reduction Reaction in Alkaline Media. *Small* **2017,** *13* (15).

2. Hwang, S. J.; Kim, S. K.; Lee, J. G.; Lee, S. C.; Jang, J. H.; Kim, P.; Lim, T. H.; Sung, Y. E.; Yoo, S. J., Role of electronic perturbation in stability and activity of Pt-based alloy nanocatalysts for oxygen reduction. *J Am Chem Soc* **2012,** *134* (48), 19508-11.

3. Mun, B. S.; Lee, C.; Stamenkovic, V.; Markovic, N. M.; Ross, P. N., Electronic structure of Pd thin films on Re(0001) studied by high-resolution core-level and valence-band photoemission. *Physical Review B* **2005,** *71* (11).

4. Stamenkovic, V. R.; Fowler, B.; Mun, B. S.; Wang, G.; Ross, P. N.; Lucas, C. A.; Markovic, N. M., Improved oxygen reduction activity on Pt3Ni(111) via increased surface site availability. *Science* **2007,** *315* (5811), 493-7.

5. Lin, L.; Xu, Y.; Han, Y.; Xu, R.; Wang, T.; Sun, Z.; Yan, Z., Spin-Magnetic Effect of d-pi Conjugation Polymer Enhanced O-H Cleavage in Water Oxidation. *J Am Chem Soc* **2024,** *146* (11), 7363-7372.

6. Zhang, H.; Chen, H. C.; Feizpoor, S.; Li, L.; Zhang, X.; Xu, X.; Zhuang, Z.; Li, Z.; Hu, W.; Snyders, R.; Wang, D.; Wang, C., Tailoring Oxygen Reduction Reaction Kinetics of Fe-N-C Catalyst via Spin Manipulation for Efficient Zinc-Air Batteries. *Adv Mater* **2024,** *36* (25), e2400523.

7. Yang, G.; Zhu, J.; Yuan, P.; Hu, Y.; Qu, G.; Lu, B. A.; Xue, X.; Yin, H.; Cheng, W.; Cheng, J.; Xu, W.; Li, J.; Hu, J.; Mu, S.; Zhang, J. N., Regulating Fe-spin state by atomically dispersed Mn-N in Fe-N-C catalysts with high oxygen reduction activity. *Nat Commun* **2021,** *12* (1), 1734.

8. Zhang, J.; Li, F.; Liu, W.; Wang, Q.; Li, X.; Hung, S. F.; Yang, H.; Liu, B., Modulating Spin of Atomic Manganese Center for High-Performance Oxygen Reduction Reaction. *Angew Chem Int Ed Engl* **2024,** *63* (51), e202412245.

9. Goerigk, L.; Grimme, S., A thorough benchmark of density functional methods for general main group thermochemistry, kinetics, and noncovalent interactions. *Phys Chem Chem Phys* **2011,** *13* (14), 6670-88.

10. Liu, M.; Liu, S.; Cui, C. X.; Miao, Q.; He, Y.; Li, X.; Xu, Q.; Zeng, G., Construction of Catalytic Covalent Organic Frameworks with Redox-Active Sites for the Oxygen Reduction and the Oxygen Evolution Reaction. *Angew Chem Int Ed Engl* **2022,** *61* (49), e202213522.

11. Yang, S.; Li, X.; Tan, T.; Mao, J.; Xu, Q.; Liu, M.; Miao, Q.; Mei, B.; Qiao, P.; Gu, S.; Sun, F.; Ma, J.; Zeng, G.; Jiang, Z., A fully-conjugated covalent organic framework-derived carbon supporting ultra-close single atom sites for ORR. *Applied Catalysis B: Environmental* **2022,** *307.*

12. Chang, Y.; Lin, C.; Wang, H.; Wu, X.; Zou, L.; Shi, J.; Xiao, Q.; Xu, Q.; Li, X.; Luo, W., Catalytic Edges in One-Dimensional Covalent Organic Frameworks for the Oxygen Reduction Reaction. *Angew Chem Int Ed Engl* **2025,** *64* (2), e202414075.

13. Yang, S.; He, Z.; Li, X.; Mei, B.; Huang, Y.; Xu, Q.; Jiang, Z., In/Outside Catalytic Sites of the Pore Walls in One-Dimensional Covalent Organic Frameworks for Oxygen Reduction Reaction. *Angew Chem Int Ed Engl* **2024**, e202418347.
14. Park, J. H.; Lee, C. H.; Ju, J. M.; Lee, J. H.; Seol, J.; Lee, S. U.; Kim, J. H., Bifunctional Covalent Organic Framework-Derived Electrocatalysts with Modulated p-Band Centers for Rechargeable Zn–Air Batteries. *Advanced Functional Materials* **2021,** *31* (25).
15. Mei, Z. Y.; Zhao, G.; Xia, C.; Cai, S.; Jing, Q.; Sheng, X.; Wang, H.; Zou, X.; Wang, L.; Guo, H.; Xia, B. Y., Regulated High-Spin State and Constrained Charge Behavior of Active Cobalt Sites in Covalent Organic Frameworks for Promoting Electrocatalytic Oxygen Reduction. *Angew Chem Int Ed Engl* **2023,** *62* (27), e202303871.
16. Wang, W.; Zhang, L.; Wang, T.; Zhang, Z.; Wang, X.; Cheng, C.; Liu, X., Inner-pore reduction nucleation of palladium nanoparticles in highly conductive wurster-type covalent organic frameworks for efficient oxygen reduction electrocatalysis. *Journal of Energy Chemistry* **2023,** *77*, 543-552.
17. Lin, C.; Yang, X.; Zhai, L.; An, S.; Ma, H.; Fu, Y.; Han, D.; Xu, Q.; Huang, N., Synergistic Modulating Interlayer Space and Electron-Transfer of Covalent Organic Frameworks for Oxygen Reduction Reaction. *Small* **2024,** *20* (28), e2308143.
18. Sun, L.; Liu, B., Mesoporous PdN Alloy Nanocubes for Efficient Electrochemical Nitrate Reduction to Ammonia. *Adv Mater* **2023,** *35* (1), e2207305.
19. Tang, J.; Liang, Z.; Qin, H.; Liu, X.; Zhai, B.; Su, Z.; Liu, Q.; Lei, H.; Liu, K.; Zhao, C.; Cao, R.; Fang, Y., Large-area Free-standing Metalloporphyrin-based Covalent Organic Framework Films by Liquid-air Interfacial Polymerization for Oxygen Electrocatalysis. *Angew Chem Int Ed Engl* **2023,** *62* (1), e202214449.
20. Wang, Y.; Nong, W.; Gong, N.; Salim, T.; Luo, M.; Tan, T. L.; Hippalgaonkar, K.; Liu, Z.; Huang, Y., Tuning Electronic Structure and Composition of FeNi Nanoalloys for Enhanced Oxygen Evolution Electrocatalysis via a General Synthesis Strategy. *Small* **2022,** *18* (41), e2203340.
21. Blochl, P. E., Projector augmented-wave method. *Phys Rev B Condens Matter* **1994,** *50* (24), 17953-17979.
22. Liu, C.; Liu, F.; Li, H.; Chen, J.; Fei, J.; Yu, Z.; Yuan, Z.; Wang, C.; Zheng, H.; Liu, Z.; Xu, M.; Henkelman, G.; Wei, L.; Chen, Y., One-Dimensional van der Waals Heterostructures as Efficient Metal-Free Oxygen Electrocatalysts. *ACS Nano* **2021,** *15* (2), 3309-3319.
23. Ju, J. M.; Lee, C. H.; Park, J. H.; Lee, J. H.; Lee, H.; Shin, J. H.; Kwak, S. Y.; Lee, S. U.; Kim, J. H., Structural and Electronic Modulations of Imidazolium Covalent Organic Framework-Derived Electrocatalysts for Oxygen Redox Reactions in Rechargeable Zn-Air Batteries. *ACS Appl Mater Interfaces* **2022,** *14* (21), 24404-24414.
24. Liao, L.; Wang, R.; Zhang, Z.; Zhang, J.; Huang, S.; Xie, W.; Wang, Y.; Xue, M.; Fang, Q.; Qiu, S., Three-dimensional porphyrin-based covalent organic frameworks as bifunctional electrocatalysts for oxygen reduction and evolution reactions. *Inorganic Chemistry Frontiers* **2025,** *12* (5), 1881-1889.

25. Wang, M.; Wang, C.; Liu, J.; Rong, F.; He, L.; Lou, Y.; Zhang, Z.; Du, M., Efficient Ag/Ag2O-Doped Cobalt Metallo-Covalent Organic Framework Electrocatalysts for Rechargeable Zinc-Air Battery. *ACS Sustainable Chemistry & Engineering* **2021,** *9* (17), 5872-5883.
26. Kang, H.; Gao, W.; Zhong, M.; Feng, Q.; Han, L.; Guo, X.; Su, B.; Lei, Z., 4,4-Biphenyldinitrile covalent triazine framework CFT-2 loaded bimetallic oxides as effective electrocatalysts. *International Journal of Hydrogen Energy* **2025,** *150.*
27. Liu, J.; Wang, C.; Song, Y.; Zhang, S.; Zhang, Z.; He, L.; Du, M., Two-dimensional triazine-based porous framework as a novel metal-free bifunctional electrocatalyst for zinc-air batty. *J Colloid Interface Sci* **2021,** *591*, 253-263.
28. Zhuang, Y.; Sun, Y.; Wang, L.; Sun, P.; Wang, J.; Zhang, P.; Li, Z., Full conjugated poly(1,4-phenyldiazo porphyrin cobalt) covalent organic framework with D-π-A structure enhancing bifunctional oxygen catalytic performance. *Journal of Energy Chemistry* **2025,** *104*, 214-224.
